# *Lactobacillus rhamnosus* GG triggers intestinal epithelium injury in zebrafish revealing host dependent beneficial effects

**DOI:** 10.1002/imt2.181

**Published:** 2024-03-25

**Authors:** Zhen Zhang, Hong‐Ling Zhang, Da‐Hai Yang, Qiang Hao, Hong‐Wei Yang, De‐Long Meng, Willem Meindert de Vos, Le‐Luo Guan, Shu‐Bin Liu, Tsegay Teame, Chen‐Chen Gao, Chao Ran, Ya‐Lin Yang, Yuan‐Yuan Yao, Qian‐Wen Ding, Zhi‐Gang Zhou

**Affiliations:** ^1^ Key Laboratory for Feed Biotechnology of the Ministry of Agriculture and Rural Affairs, Institute of Feed Research Chinese Academy of Agricultural Sciences Beijing China; ^2^ Faculty of Land and Food Systems The University of British Columbia Vancouver Canada; ^3^ China‐Norway Joint Lab on Fish Gut Microbiota, Institute of Feed Research Chinese Academy of Agricultural Sciences Beijing China; ^4^ State Key Laboratory of Bioreactor Engineering East China University of Science and Technology Shanghai China; ^5^ Laboratory of Microbiology Wageningen University and Research Wageningen Netherlands; ^6^ Human Microbiome Research Program, Faculty of Medicine University of Helsinki Helsinki Finland; ^7^ Tigray Agricultural Research Institute Mekelle Ethiopia

**Keywords:** intestinal mucosal damage, *Lactobacillus rhamnosus* GG, probiotic safety, SpaC, zebrafish

## Abstract

*Lactobacillus rhamnosus* GG (LGG), the well‐characterized human‐derived probiotic strain, possesses excellent properties in the maintenance of intestinal homeostasis, immunoregulation and defense against gastrointestinal pathogens in mammals. Here, we demonstrate that the SpaC pilin of LGG causes intestinal epithelium injury by inducing cell pyroptosis and gut microbial dysbiosis in zebrafish. Dietary SpaC activates Caspase‐3−GSDMEa pathways in the intestinal epithelium, promotes intestinal pyroptosis and increases lipopolysaccharide (LPS)‐producing gut microbes in zebrafish. The increased LPS subsequently activates Gaspy2−GSDMEb pyroptosis pathway. Further analysis reveals the Caspase‐3−GSDMEa pyroptosis is initiated by the species‐specific recognition of SpaC by TLR4ba, which accounts for the species‐specificity of the SpaC‐inducing intestinal pyroptosis in zebrafish. The observed pyroptosis‐driven gut injury and microbial dysbiosis by LGG in zebrafish suggest that host‐specific beneficial/harmful mechanisms are critical safety issues when applying probiotics derived from other host species and need more attention.

## Highlights

- SpaC pilin acts as a causative factor of *Lactobacillus rhamnosus* GG‐induced intestinal mucosa damage in zebrafish.
- Dietary SpaC directly induces intestinal pyroptosis by activating GSDMEa in zebrafish.
- Dietary SpaC induces gut microbial dysbiosis characterized by higher abundance of lipopolysaccharide (LPS)‐producing gut microbes in zebrafish.
- LPS‐producing gut microbes subsequently activate Gaspy2−GSDMEb pyroptosis pathway in zebrafish.

## Graphical abstract

*Lactobacillus rhamnosus* GG (LGG) is a well‐studied human‐derived probiotic strain. It possesses excellent properties in the maintenance of intestinal homeostasis, immunoregulation and defense against gastrointestinal pathogens in mammals. Zhang et al. report that SpaC pilin of LGG causes intestinal epithelium injury by inducing cell pyroptosis and gut microbial dysbiosis in zebrafish, indicating safety issues of probiotics derived from other species of the host when administrated to aquaculture.

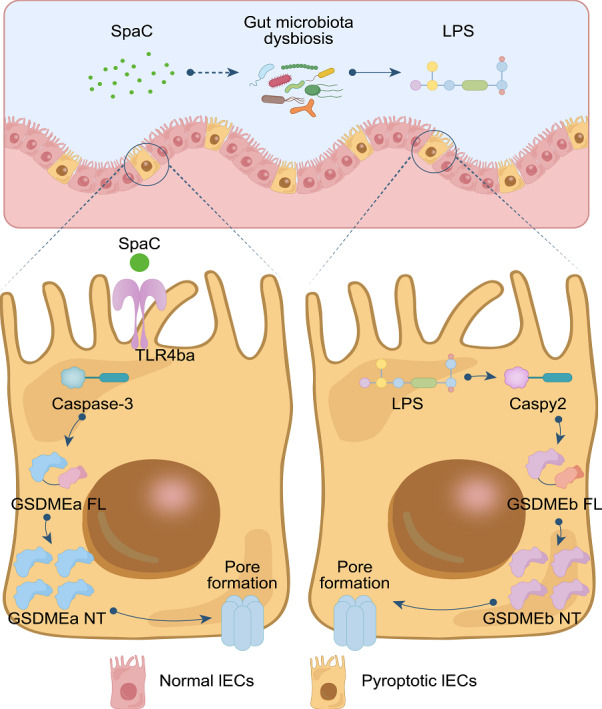

## INTRODUCTION

Probiotics are widely used in humans and farmed animals [1]. As recommended by the Food and Agriculture Organization of the United Nations/World Health Organization, the conventional source of probiotics for human use is the organisms originated from the human gastrointestinal tract [2]. Besides, bacteria isolated from new sources like fermented products of animal origin are also currently being proposed for use as human probiotics [2]. Meanwhile, many human‐derived probiotics are applied to farmed animals including poultry, pigs and ruminants. There is an increasing inclination for the application of terrestrial‐derived probiotics to aquatic animals [3, 4]. However, it is poorly defined on that whether nonhost‐origin probiotics can be used throughout humans and food‐producing animals without causing safety concerns. Especially, there are no globally harmonized rules or regulations about the safety of probiotics in animal feed and human food [5], and no safety data were reported [6].

*Lactobacillus rhamnosus* GG (LGG), a human‐derived probiotic strain, favorably maintains intestinal homeostasis, modulates immune response and resists infection of gastrointestinal pathogens in mammals [7, 8, 9]. The surface adhesive protein polymers of LGG, called “pilus”, bind to the epithelial extracellular matrix proteins and mucus, contributing to the mucosal adherence of LGG [10, 11, 12, 13]. LGG pilus is composed of three pilin subunits including SpaC, SpaB and SpaA pilin subunits [11], which mediate the immunomodulatory interactions with intestinal epithelial cells (IECs) [14]. It has been reported that SpaC pilin subunits act as adhesion factors, and can bind to the mucosal surface [15]. Thus nowadays, health‐beneficial effects of LGG have made it become one of the widely used probiotics in human and several food‐producing animal species.

Zebrafish (*Danio rerio*), which shares approximately 70% of homologous genes with human [16], is an efficient and important nonmammalian model with many advantages in the studies of host‐microbe interaction [17, 18], toll‐like receptor (TLR) signaling pathway [19], and adaptive immune system [20]. Germ‐free (GF) zebrafish model has been developed as an essential tool for studying host‐microbe interaction [18]. Zebrafish has been extensively used as an alternative model organism to assess the effectiveness of different probiotics [21]. In our previous study, we found that LGG did not enhance the resistance to aquatic pathogen infection (*Aeromonas hydrophila* NJ‐1) in zebrafish [22]. Moreover, apparent morphological damage was observed in the intestines of zebrafish immersed with 10^7^ colony forming units (CFUs)/mL LGG for 14 days [22]. We speculated that a host specificity exists in zebrafish in term of pathogen‐resistant effect and intestinal health in response to LGG. In addition, alternative pattern recognition receptors may be involved with this host specificity effect of LGG in zebrafish.

Recent studies revealed that pro‐inflammatory pathways of programmed cell death, especially pyroptosis, participated in IECs' death [23]. Pyroptosis is widely observed in vertebrates, including mammals and teleost [24]. It is characterized by cell lysis and release of pro‐inflammatory cytokines which are caused by damage‐associated molecular patterns and pathogen‐associated molecular patterns [25]. Pyroptosis is suggested to be strongly regulated by inflammasome activation [26] with the mature form of interleukin‐1β (IL‐1β), which is usually used as an indicator of pyroptosis [27, 28]. Inflammasomes are formed upon bacterial infection and induce autoactivation of cysteinyl aspartate specific proteinases (caspases) [29]. Activated caspases cleave gasdermins (GSDMs) to produce N‐terminal fragments which then lead to the formation of pores, eventually resulting in pyroptosis [29]. Therefore, pyroptosis plays an important role in antimicrobial host defense by removing damaged cells [30]. But over‐activation of pyroptosis is detrimental to tissue integrity and even causes host death [31].

We speculated that pyroptosis may participate in the host‐specific effect of LGG on zebrafish. However, only a pair of GSDME (GSDMEa/b) was identified in zebrafish [32, 33, 34, 35]. GSDMEa of zebrafish can switch tumour necrosis factor (TNF)‐induced apoptosis to pyroptosis in Hela cells, and the Caspase‐3−cleavable site exists in the GSDMEa of zebrafish [32]. The noncanonical inflammasome Caspy2−GSDMEb pathway of pyroptosis was demonstrated in zebrafish in vivo [33, 34], however the evidence of pyroptotic pathway mediated by Caspase‐3−GSDMEa is still lacking in zebrafish. Therefore, in the present study we evaluated the species‐specific effect and elucidated the mode of actions of SpaC pilin subunit in LGG on the intestinal health in zebrafish using germ‐free zebrafish, microbiota‐conserved zebrafish and cell models.

## RESULTS

### SpaC pilin subunit is responsible for zebrafish intestinal mucosa damage

**Figure 1:**
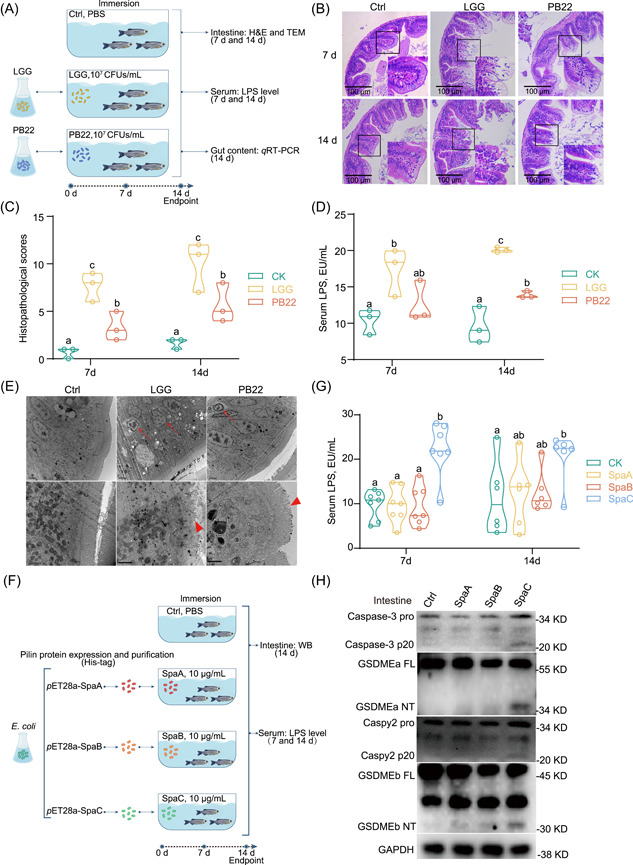
SpaC pilin subunit is responsible for zebrafish intestinal mucosa damage. (A) One‐month old zebrafish were immersed with either LGG or PB22 at 10^7^ CFUs/mL for 7 or 14 days. (B) Representative intestinal histology images by H&E staining. Scale bar 100 μm. (C) Total histological score measuring the severity of the intestinal injury at 7 and 14 days (Day 7: *F*_2,6_ = 16.545; Day 14: *F*_2,6_ = 13.971; *n* = 3). Quantitative data was obtained by the indicators including: disorganized microvilli, edema or inflammatory infiltrate in lamina propria, vacuolar degeneration of IECs, cell shedding and necrosis. (D) Serum LPS levels (Day 7: *F*_2,6_ = 5.185; Day 14: *F*_2,6_ = 37.515; *n* = 3, pool of three zebrafish per sample). (E) Representative electron micrographs of intestinal sections at Day 14. Scale bar 2 μm (up). Scale bar 1 μm (below). Arrows indicate dying or dead intestinal epithelial cells. Triangular arrows indicate microvilli damage. (F) One‐month old zebrafish were immersed with recombinant SpaA, SpaB, or SpaC at concentrations of 10 μg/mL for Day 7 or 14, respectively. (G) Serum LPS levels (Day 7: *F*_3,24_ = 13.562; Day 14: *F*_3,20_ = 2.543; *n* = 6 or 7, pool of 3 zebrafish per sample). (H) A representative western blot analysis showing Caspase‐3 activation, GSDMEa cleavage, Caspy2 activation and GSDMEb cleavage. Numbers of biologically independent samples are labeled on the violin plots. Horizontal line represents median in the violin plots. Statistics: one‐way ANOVA followed by Duncan's test. Treatments in plots labeled with different letters on top represent statistically significant results (*p* < 0.05). Caspase, cysteinyl aspartate specific proteinase; CFUs, colony forming units; GSDM, gasdermin; H&E, Hematoxylin and eosin; IECs, intestinal epithelial cells; LGG, *Lactobacillus rhamnosus* GG; LPS, lipopolysaccharide.

One‐month old zebrafish were immersed with either wild‐type (WT) LGG or PB22 at 10^7^ CFUs/mL for 7 or 14 d (Figure 1A). The immersion of LGG induced apparent intestinal histopathological damage (Figure 1B,C, Table S1), and significantly elevated serum lipopolysaccharide (LPS) levels in zebrafish (Figure 1D). When PB22, a mutant strain of LGG, which lacks SpaCBA pilus, was administrated to zebrafish, the intestinal injury was much slighter than that induced by the WT LGG (Figure 1B,C), and the level of serum LPS in PB22‐treated zebrafish was significantly lower than that in WT LGG‐treated zebrafish (Figure 1D). Furthermore, the LGG‐treated zebrafish showed IECs with abnormal nuclei characterized by chromatic agglutination and karyopyknosis with or without nuclear membrane blebbing on the intestinal epithelial layer, indicating the dying or death of IECs (Figure 1E). These findings suggest the intestinal mucosa‐damaging effect of SpaCBA pilus in zebrafish. In this study, in both LGG and SpaC‐immersed zebrafish, no mobility dysfunctions and traumatic symptoms were observed in the fish.

To define which pilin subunit of SpaCBA pilus is responsible for the intestinal mucosa damage, recombinant SpaA, SpaB or SpaC was purified and immersed to zebrafish (Figures 1F, S1A). Similar to that observed in LGG‐immersed zebrafish, the level of serum LPS was significantly increased in SpaC‐immersed zebrafish, but not in SpaA‐ or SpaB‐immersed zebrafish (Figure 1G). These results suggest that SpaC pilin subunit is responsible for the intestinal mucosa‐damaging of LGG in zebrafish.

To determine whether IECs die for pro‐inflammatory pyroptosis upon LGG treatment, we first detected the form of IL‐1β in the intestine. The mature form of IL‐1β was observed in the intestine of LGG‐immersed zebrafish (Figure S1B). This result indicates that LGG treatment may activate inflammasomes. Furthermore, the cleavages of Caspase‐3, GSDMEa‐N, Caspy2 and GSDMEb‐N were observed in SpaC‐immersed zebrafish (Figure 1H). These results collectively suggest that pyroptosis participates in the intestinal mucosa‐damaging effect of SpaC pilin subunit in zebrafish.

### Dietary SpaC induces intestinal pyroptosis and gut microbial dysbiosis in zebrafish

**Figure 2:**
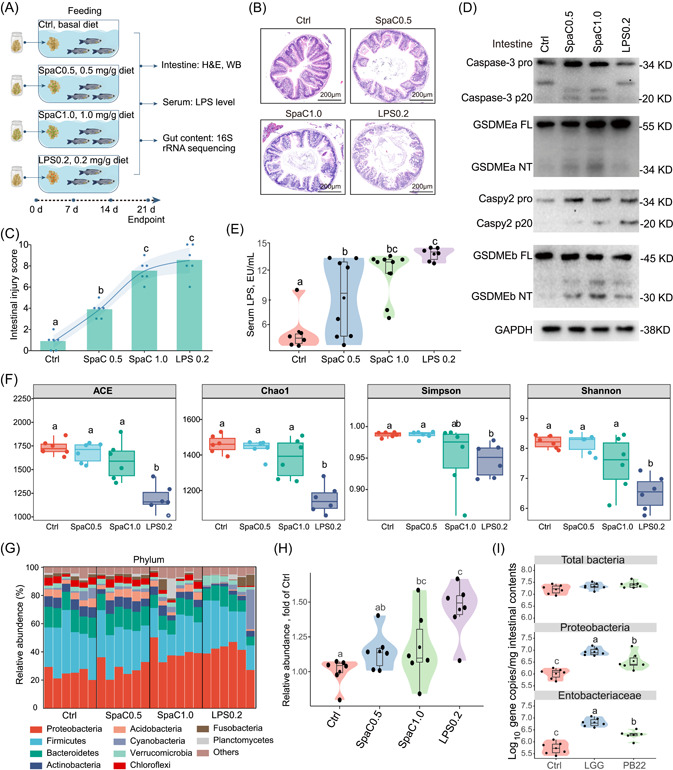
Dietary SpaC and LPS induce intestinal pyroptosis and gut microbial dysbiosis in zebrafish. (A) One‐month old zebrafish were fed with diets supplemented with 0.5 and 1.0 mg/g SpaC or diet supplemented with 0.2 mg/g LPS for three weeks. (B) Representative intestinal histology images by H&E staining. (C) Total histological score measuring the severity of the intestinal injury (Values are means. *F*_3,20_ = 65.490; *n* = 6). (D) A representative western blot analysis showing Caspase‐3 activation, GSDMEa cleavage, Caspy2 activation and GSDMEb cleavage. (E) Serum LPS levels (*F*_3,25_ = 9.826; *n* = 6−9, pool of three zebrafish per sample). (F) Indexes of α diversity (ACE: *F*_3,20_ = 17.869; Chao1: *F*_3,20_ = 20.649; Simpson: *F*_3,20_ = 3.273; Shannon: *F*_3,20_ = 11.611; *n* = 6, pool of 6 zebrafish per sample). (G) The composition of gut microbiota at phylum level (*n* = 6, pool of six zebrafish per sample). (H) The relative abundance of bacteria producing LPS (*F*_3,20_ = 5.907; *n* = 6). (I) Total number of bacteria (*F*_3,20_ = 2.332), the number of Proteobacteria (*F*_3,20_ = 18.276) and the number of *Entobacteriaceae* (*F*_2,14_ = 22.373) in the intestinal microbiota of one‐month old zebrafish immersed with either LGG or PB22 at 10^7^ CFUs/mL for Day 14 (*n* = 5−6). The Numbers of biologically independent samples are labeled on the violin plots. Box represents median ± interquartile range, whiskers represent 1.5× interquartile range. Statistics: one‐way ANOVA followed by Duncan's test. Treatments in violin or box plots labeled with different letters on top represent statistically significant results (*p* < 0.05).

Generally, oral administration is regarded as the most widely used method for probiotics application [36]. Thus, we designed a 3‐week feeding trial with SpaC‐supplemented diets to further validate the effect of SpaC on zebrafish intestinal pyroptosis (Figure 2A). Due to the higher level of serum LPS in SpaC‐immersed zebrafish, an experimental group fed with the LPS‐supplemented diet (LPS: 0.2 mg/g diet) was also included (Figure 2A, Table S2). Consistent with SpaC immersion, apparent histopathological characteristics of intestinal injury were observed in both the SpaC‐fed and LPS‐fed zebrafish (Figure 2B,C, Table S3). Thus, both diets containing SpaC and LPS can lead to intestinal epithelium damage. Furthermore, immunoblotting analysis revealed the occurrence of cleavage of Caspase‐3, GSDMEa, Caspy2 and GSDMEb in the intestine of SpaC‐fed zebrafish (Figure 2D), whereas only cleavages of Caspy2 and GSDMEb were observed in the intestine of LPS‐fed zebrafish (Figure 2D). These results suggest that SpaC can activate Caspase‐3−GSDMEa and Caspy2−GSDMEb pyroptosis pathways, while LPS can only activate Caspy2–GSDMEb pyroptosis pathway in the intestinal mucosa of zebrafish.

Compared to zebrafish fed with the control (Ctrl) diet, the level of serum LPS significantly increased in SpaC‐fed zebrafish (Figure 2E). As the elevation of serum LPS is related to the outgrowth of Proteobacteria members [37], we detected the composition of gut microbiota by 16S ribosomal ribonucleic acid (rRNA) sequencing technique. The differences in species diversity and composition of gut microbiota among zebrafish fed the Ctrl, SpaC0.5, SpaC1.0 and LPS0.2 diets were revealed by the rarefaction curves and principal coordinate analysis (Figures 2F, S2A,B). The changes in gut microbiota composition were further supported by the significant increase in the relative abundance of Proteobacteria in both the SpaC1.0 (39.36%) and LPS0.2 groups (39.99%) versus the Ctrl group (24.60%) (Figure 2G). Accordingly, the abundance of *Enterobacteriaceae* was significantly increased in the SpaC1.0 group, and the abundance of *Sporomusa*, *Acinetobacter*, *Desulfovibrio* and *Peptococcaceae* were significantly increased in the LPS group (Figure S2C,D). According to Kyoto Encyclopedia of Genes and Genomes (KEGG) pathways analysis, the operational taxonomic units (OTUs) that are capable of producing LPS were significantly increased in the SpaC1.0 and LPS groups (Figure 2H). These results suggest that both diets containing SpaC and LPS can lead to gut microbiota dysbiosis and elevation of serum LPS.

In addition, similar changes in gut microbiota were observed in LGG‐ and PB22‐immersed zebrafish (Figure 2I). With no influence on the total number of bacteria, LGG immersion altered the gut microbiota by increasing the relative abundance of Proteobacteria significantly as compared to the Ctrl group (Figure 2I). At the genus level, a significantly higher abundance of *Entobacteriaceae* was observed in LGG immersion group (Figure 2I). Though there was an increase in the abundance of Proteobacteria and *Entobacteriaceae* in the PB22 immersed group, it was notably lower compared with LGG‐immersed group (Figure 2I). These results suggest that both LGG‐immersion and SpaC‐feeding treatments can alter gut microbiota composition in a similar manner, indicating that SpaC has a contribution in alteration of intestinal microbiota of zebrafish.

### Gut microbiota partly accounts for the intestinal pyroptosis induced by the SpaC‐containing diet

**Figure 3:**
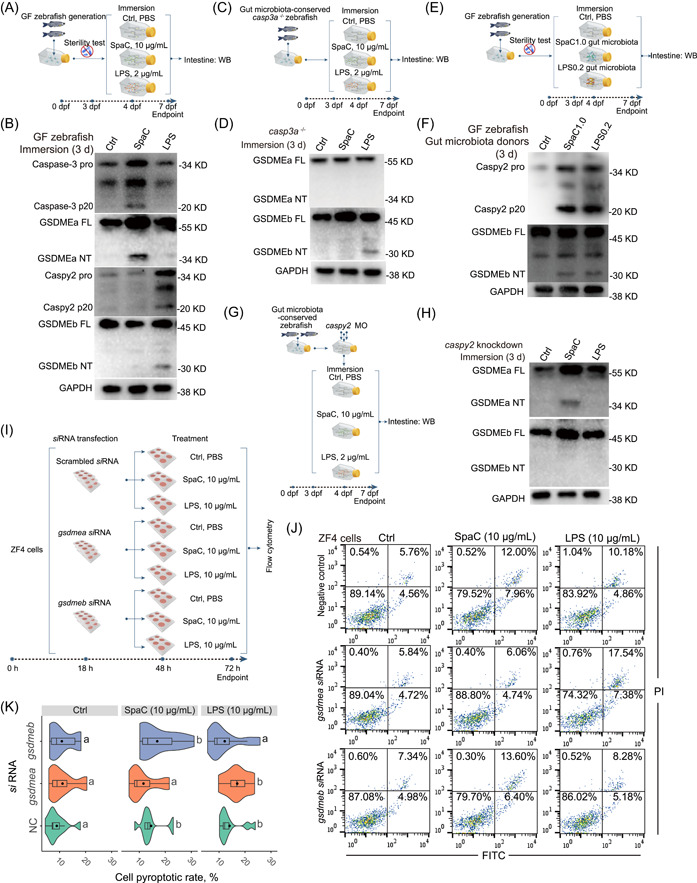
SpaC and LPS induce pyroptosis in gut microbiota‐independent and ‐dependent manners. (A) GF zebrafish (4‐dpf) were immersed with 10 μg/mL SpaC or LPS for 3 d. (B) A representative western blot analysis showing Caspase‐3 activation, GSDMEa cleavage, Caspy2 activation and GSDMEb cleavage in GF zebrafish. (C) The *casp3a* ^−/−^ zebrafish (4‐dpf) were immersed with 10 μg/mL SpaC or LPS for Day 3. (D) A representative western blot analysis showing GSDMEa cleavage and GSDMEb cleavage in *casp3a* ^−/−^ zebrafish. (E) Gut microbiota of zebrafish fed with SpaC or LPS diet was transferred to GF zebrafish for Day 3. (F) A representative western blot analysis showing Caspy2 activation and GSDMEb cleavage in GF zebrafish received the gut microbiota from one‐month old zebrafish fed the SpaC1.0 or LPS0.2 diet for 3 weeks. (G) Zebrafish (4‐dpf) with *caspy2* knockdown were immersed with 10 μg/mL SpaC or LPS for Day 3. (H) A representative western blot analysis showing GSDMEa cleavage and GSDMEb cleavage in *caspy2* knockdown zebrafish. (I) ZF4 cells were treated with 10 μg/mL SpaC or LPS when gene encoding *GSDMEa* or *GSDMEb* were silenced. (J) Representative plots of cell pyroptotic/apoptotic rate. (K) Statistical pyroptotic rates at 24 h posttreatment (Scrambled *si*RNA: *F*_2,27_ = 4.954; *gsdmea*: *F*_2,27_ = 4.067; *gsdmeb*: *F*_2,27_ = 2.449; *n* = 10). Box represents median ± interquartile range, whiskers represent 1.5× interquartile range in the violin plots. Statistics: Student's *t*‐test. Treatments in plots labeled with different letters on top represent statistically significant results (*p* < 0.05). dpf, day postfertilization; GF, germ‐free; *si*RNA, specific small interfering RNA.

To investigate the role of gut microbiota in the intestinal pyroptosis process induced by SpaC, GF zebrafish were immersed with 10 μg/mL SpaC or 2 μg/mL LPS for 3 d (Figure 3A). Different from the results observed in SpaC‐fed gut microbiota‐conserved zebrafish, only Caspase‐3 and GSDMEa were cleaved in SpaC‐immersed GF zebrafish (Figure 3B). With no involvement of gut microbiota, SpaC should only activate Caspase‐3−GSDMEa pathway and had no influence on Caspy2−GSDMEb pathway (Figure 3B). However, the cleaved forms of Caspy2 and GSDMEb were still observed in LPS‐immersed GF zebrafish (Figure 3B). These results suggest the important role of gut microbiota in the activation of Caspy2−GSDMEb pathway.

To determine whether there is a link between the activation of Caspase‐3−GSDMEa and Caspy2−GSDMEb pathways in zebrafish, gut microbiota‐conserved *casp3a* ^−/−^ zebrafish were immersed with 10 μg/mL SpaC or LPS for 3 d (Figure 3C). With the knockout of *caspase‐3a*, SpaC immersion did not activate the cleavages of GSDMEa as well as GSDMEb (Figure 3D). These results suggest that Caspase‐3a is essential for the cleavages of GSDMEa and GSDMEb in gut microbiota‐conserved zebrafish when treated by SpaC.

Then the role of gut microbiota from SpaC‐ or LPS‐fed zebrafish was defined by using the GF zebrafish‐gut microbiota inoculation model (Figure 3E). The gut microbiota from SpaC‐ or LPS‐fed zebrafish led to the cleavages of Caspy2 and GSDMEb (Figure 3F). These results suggest that gut microbiota induced by the SpaC‐containing diet can activate intestinal pyroptosis at least through the pathway of Caspy2−GSDMEb.

In contrast to WT zebrafish under gut microbiota‐conserved conditions, *caspy2* knockdown merely abolished the cleavage of GSDMEb caused by SpaC or LPS immersion, but with no influence on SpaC‐induced GSDMEa cleavage (Figure 3G,H). These results suggest that Caspy2 is merely essential for the cleavage of GSDMEb but not GSDMEa in SpaC‐treated zebrafish.

To validate the essentiality of GSDMEa and GSDMEb in cell pyroptosis induced by SpaC or LPS, *gsdmea* and *gsdmeb* were knocked down by transfected zebrafish fibroblast cells (ZF4) with specific small interfering RNAs (*si*RNAs), respectively (Figures 3I, S3A,B). At 24 h posttransfection, ZF4 cells were treated with 10 μg/mL SpaC or LPS for an additional 24 h. The knockdown of *gsdmea* significantly reduced SpaC‐induced cell pyroptosis in ZF4 cells, while the knockdown of *gsdmeb* significantly reduced LPS‐induced cell pyroptosis in ZF4 cells (Figure 3J,K). Additionally, no significant increase in cell apoptotic rate was observed when *gsdmea* or *gsdmeb* was knocked down in ZF4 cells (Figures 3K, S3C). These results further validate that the GSDMEa and GSDMEb are essential for cell pyroptosis induced by SpaC and LPS in zebrafish, respectively.

### The interaction between SpaC and zebrafish toll‐like receptor 4ba (TLR4ba) initiates intestinal pyroptosis

**Figure 4:**
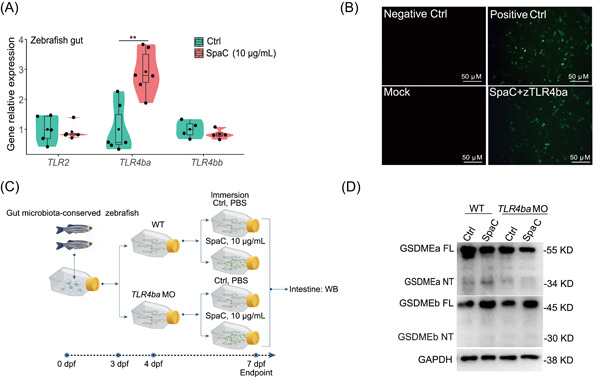
LGG SpaC pilin triggers intestinal pyroptosis by interacting with zebrafish TLR4ba. (A) One‐month old zebrafish were immersed with recombinant SpaC at concentrations of 10 μg/mL for Day 7. The relative mRNA expression of genes encoding *TLR2*, *TLR4ba* and *TLR4bb* in zebrafish intestine as measured by *q*RT‐PCR (*TLR2*: *F*_1,8_ = 0.116; *TLR4ba*: *F*_1,10_ = 18.075; *TLR4bb*: *F*_1,6_ = 0.912; *n* = 6). (B) Characterization of the interaction between SpaC and zebrafish TLR4ba by BiFC. (C) The *TLR4ba* was knocked down by using morpholino oligonucleotides in zebrafish larvae. (D) A representative western blot analysis showing GSDMEa and GSDMEb cleavage in zebrafish larvae with *TLR4ba* knockdown upon SpaC immersion. The Numbers of biologically independent samples are labeled on the violin plots. Box represents median ± interquartile range, whiskers represent 1.5× interquartile range. Statistics: Student's *t*‐test. *p* < 0.01, **. BiFC, bimolecular fluorescent complementation; *q*RT‐PCR, quantitative real‐time PCR reaction; TLR, toll‐like receptor; Negative Ctrl (nontransfection), mock (cotransfection of *p*BiFC‐VC155 and *p*BiFC‐VN173), positive Ctrl (cotransfection of *p*BiFC‐bFosVC155 and *p*BiFC‐bjunVN173), experimental group (cotransfection of *p*BiFC‐VN173‐SpaC and *p*BiFC‐VC155‐zTLR4ba).

The SpaC pilin subunit is one kind of microbe‐associated molecular pattern (MAMP) [38]. Thus, we attempted to identify the specific receptor that can recognize SpaC in the zebrafish gut. The recombinant His‐tagged SpaC protein was used as the bait protein to pull down its interacting proteins *ex vivo* (Figure S4A). Then these interacting protein complexes were subject to liquid chromatography−mass spectrometry (LC−MS)/MS analysis (Figure S4B). A total of 685 potential interacting proteins were identified by LC−MS/MS analysis (Table S4). The suspected membrane receptors are listed in Figure S4C, including TLR4ba. Consistently, *TLR4ba* was significantly upregulated in the intestine of zebrafish when immersed with recombinant SpaC for 7 d (Figures 4A, S4D). The interaction between TLR4ba and SpaC was further demonstrated by the formation of the bimolecular fluorescent complex of SpaC and the extracellular domain of zebrafish TLR4ba (zTLR4ba) in human embryonic kidney (HEK293) cells via bimolecular fluorescence complementation (BiFC) (Figure 4B). Besides, the cleavage of GSDMEa caused by SpaC immersion was abolished by *TLR4ba* knockdown in GF zebrafish (Figure 4C,D). These results suggested that TLR4ba is essential for SpaC‐induced pyroptosis.

### The comparative TLR signaling to SpaC in zebrafish and human

**Figure 5:**
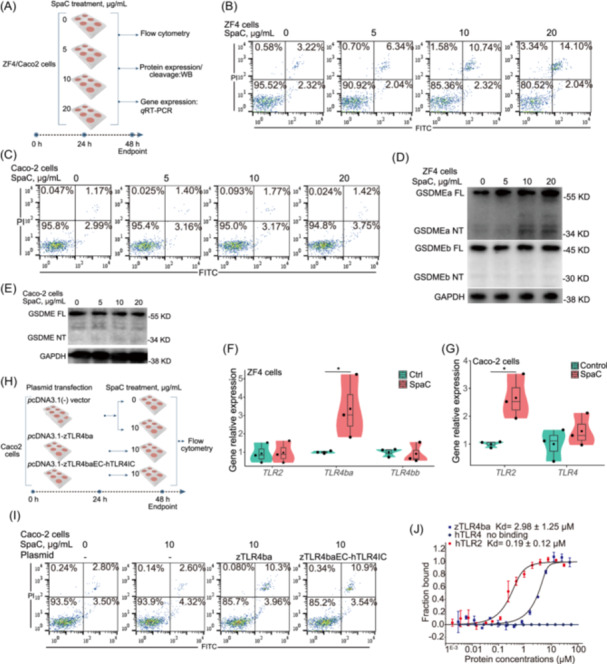
SpaC induces pyroptosis in zebrafish ZF4 cells rather than in human Caco‐2 cells. (A) Cells were treated with increasing concentrations of SpaC (0, 5, 10 and 20 μg/mL) for 24 h. (B) The flow cytometry results of ZF4 cells treated with recombinant SpaC protein in different dose for 24 h. (C) The flow cytometry results of Caco‐2 cells treated with recombinant SpaC protein in different dose for 24 h. (D) A representative western blot analysis showing GSDMEa and GSDMEb cleavage in ZF4 cells. (E) A representative western blot analysis showing GSDME cleavage in Caco‐2 cells. (F) The relative mRNA expression of genes encoding *TLR2*, *TLR4ba* and *TLR4bb* in ZF4 cells (*TLR2*: *F*_1,4_ = 0.024; *TLR4ba*: *F*_1,4_ = 5.550; *TLR4bb*: *F*_1,4_ = 0.062; *n* = 3). (G) The relative mRNA expression of genes encoding *TLR2* and *TLR4* in Caco‐2 cells (*TLR2*: *F*_1,4_ = 12.387; *TLR4*: *F*_1,4_ = 0.953; *n* = 3). (H) Plasmid expressing zTLR4ba or zTLR4baEC‐hTLRIC was transfected into Caco2 cells. (I) The flow cytometry results of Caco‐2 cells or Caco‐2 cells overexpressed zTLR4ba or zTLR4baEC‐hTLRIC treated with 10 μg/mL SpaC protein for 24 h. (J) The strength of interaction (binding affinity) between purified SpaC and TLR proteins was measured by MST technique (*n* = 3). The Numbers of biologically independent samples are labeled on the violin plots. Box represents median ± interquartile range, whiskers represent 1.5× interquartile range. Statistics: Student's *t*‐test. *p* < 0.05, *. MST, MicroScale Thermophoresis; ZF4, zebrafish fibroblast cells; zTLR4ba, zebrafish TLR4ba; zTLR4baEC‐hTLRIC, the fusion of the extracellular domain of zTLR4ba and the transmembrane and intracellular domain of hTLR4.

To further investigate the differential effects of SpaC in different hosts, the zebrafish ZF4 cells and human Caco‐2 cells were treated with recombinant SpaC (Figure 5A). Recombinant SpaC increased the cell pyroptotic rate of ZF4 cells in a dose‐dependent manner but not Caco‐2 cells (Figure 5B,C). Consistently, the cleavage of GSDMEa was observed in ZF4 cells (Figure 5D), while no cleavage of GSDME was observed in Caco‐2 cells (Figure 5E) when treated with higher concentrations of SpaC. These results suggest that SpaC may induce cell pyroptosis in zebrafish but not in humans. The expression of zebrafish *TLR4ba* was significantly upregulated in SpaC‐treated ZF4 cells (Figure 5F). However, the recombinant SpaC significantly upregulated the expression of toll‐like receptor 2 (*TLR2*), but not toll‐like receptor 4 (*TLR4*) in Caco‐2 cells (Figure 5G). These results indicate that SpaC is the species‐specific ligand to zTLR4ba in zebrafish.

Sullivan et al. proposed that the shift of ligand recognition is likely due to the extracellular portions of zebrafish TLR4ba and TLR4bb, rather than the changes in the Toll/IL‐1 receptor (TIR) domains [39]. Thus, we constructed a plasmid carrying the gene encoding zTLR4ba or zTLR4baEC‐hTLRIC (the fusion of the extracellular domain of zTLR4ba and the transmembrane and intracellular domain of human TLR4 (hTLR4)) and expressed in Caco‐2 cells (Figure 5H). Compared to Caco‐2 cells transfected with the blank vector, the pyroptotic rate of Caco‐2 cells expressing *p*cDNA3.1‐zTLR4ba or *pc*DNA3.1‐zTLR4baEC‐hTLRIC was significantly increased when treated with SpaC (Figure 5I). These results suggest the contribution of the extracellular domain of zTLR4ba in the species‐specific recognition to SpaC. Additionally, the species‐specific binding of SpaC to TLRs was further validated by measuring the strength of interaction (binding affinity) between purified SpaC and TLR proteins via the MicroScale Thermophoresis (MST) technique. No affinity was observed between hTLR4 and SpaC (Figure 5J). SpaC had a higher affinity to human TLR2 (hTLR2) than zTLR4ba (Figure 5J). These results suggest that SpaC can interact with zTLR4ba and hTLR2 but not hTLR4.

## DISCUSSION

**Figure 6:**
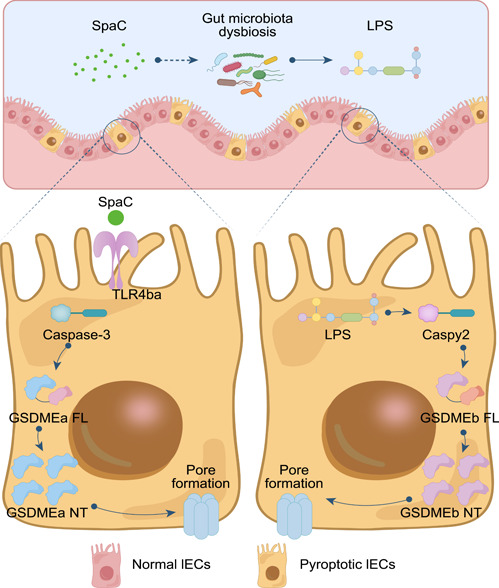
Illustration of the mechanisms underlying the activation of SpaC to intestinal pyroptosis. The SpaC pilin of LGG induces intestinal epithelium injury through a species‐specific activation of TLR4ba, which initiates Caspase‐3−GSDMEa pyroptosis and secondarily activates Gaspy2−GSDMEb pyroptosis via gut microbiota dysbiosis.

This study demonstrated the role of SpaC pilin subunit in LGG‐induced intestinal mucosa damage and identified relevant damaging pathways in zebrafish. The present study revealed the adverse effect of LGG, a widely used human‐derived probiotic strain, on the intestinal health of zebrafish from the perspective of SpaC‐induced IECs' pyroptosis and gut microbial dysbiosis (Figure 6). The species‐specific recognization of SpaC by TLR4ba was validated to be essential for the initiation of SpaC‐induced pyroptosis. The GSDMEa‐triggering pyroptosis disturbed gut microbiota. Sequentially, gut microbiota produced LPS and triggered GSDMEb pyroptosis pathway.

In this study, the different effects of LGG and PB22 on intestinal histology and serum LPS level suggest that the SpaCBA pilus is the major component that can lead to intestinal mucosa‐damaging effects in zebrafish. Furthermore, the different effects of SpaA, SpaB and SpaC pilin subunits on serum LPS levels indicate that the SpaC pilin subunit was responsible for the intestinal damage in zebrafish. The paracellular penetration of LPS through intestinal epithelium indicates disordered tight junctions and disrupted intestinal barrier [40]. Disrupted intestinal barrier is resulted from excessive epithelial cell loss and/or decreased regenerative capacity [41]. Thus, the serum LPS level was used as an indicator of intestinal barrier integrity in this study. LPS is the major component of the outer membrane of Proteobacteria [37], and constantly released to the surroundings by members of Proteobacteria in a form of outer membrane vesicles [42, 43]. Thus, in addition to being an indicator of intestinal barrier integrity, the elevation of serum LPS is also an indicator of the outgrowth of Proteobacteria members [37]. The results of 16S rRNA gene sequencing revealed gut microbiota dysbiosis induced by SpaC, which was characterized by the increasing abundance of Proteobacteria. Besides, KEGG analysis revealed the functions of LPS biosysthesis of SpaC‐induced gut microbiota were promoted. Thus, in addition to the gut leakage of LPS caused by intestinal damage, the enrichment of LPS‐producing gut microbiota may be partly related to the elevation of serum LPS when fed the SpaC‐containing diet. Similar results were observed in zebrafish immersed or fed with the LGG‐supplemented diet rather than the PB22‐supplemented diet. These results suggest that SpaC may be the key factor that mediates the influence of LGG on gut microbiota because supplementation of PB22 did not affect the gut microbiota of the fish as that of LGG. Further analysis of the Caspases and executors of Caspase‐dependent pyroptosis suggests that SpaC may be the MAMP of LGG that activates intestinal pyroptosis in zebrafish.

In mammals, oral administration of a microbial protein is finely common. For instance, feeding on a high‐fat diet supplemented with Amuc_1100, pili‐like proteins, isolated from the outer membrane of *Akkermansia muciniphila* at a level of 3 or 5 μg per day per mouse, improved the lipid metabolism of obese and diabetic mice [44, 45]. In this study, the oral administration of SpaC at a level of approximately 5 or 10 μg per day per zebrafish induced intestinal mucosa damage and pyroptosis in zebrafish. SpaC is intolerant to trypsinisation treatment and its degradation products may not necessarily possess the same activity or efficacy as the intact SpaC protein [46]. Thus, we can conclude that the degradation products of SpaC did not participate in inducing intestinal mucosa damage and pyroptosis in zebrafish. However, the apparent digestibility coefficient of protein ranges from 47%−90% in cyprinid fish [47, 48]. This means parts of SpaC can remain intact in the gastrointestinal tract and bind to intestinal mucosa to activate pyroptosis pathways.

Even though the activated pyroptosis pathways of SpaC and LPS are different, LPS also induce intestinal damage. This result was supported by the studies performed using zebrafish on the role of LPS in the activation of pyroptosis [33, 34]. Therefore, the diet containing SpaC may activate the Caspy2−GSDMEb pyroptosis pathway by LPS‐producing gut microbiota. In line with this speculation, in the absence of gut microbiota, only the Caspase‐3−GSDMEa pathway was activated by SpaC, but not Caspy2−GSDMEb pathway. The activation of Caspy2−GSDMEb pathway by LPS in GF zebrafish indirectly supports the contribution of LPS‐producing bacteria in intestinal pyroptosis induced by SpaC. This was further validated by the activation of Caspy2−GSDMEb pathway when the gut microbiota from SpaC‐fed zebrafish was transferred to GF zebrafish.

In gut microbiota‐conserved zebrafish, the knockout of *caspase‐3a* blocked the cleavage of GSDMEa, suggesting that zebrafish Caspase‐3 is essential for GSDMEa cleavage. This is in line with the function of mammalian Caspase‐3 [32]. Interestingly, the cleavage of GSDMEb was also deleted by the knockout of *caspase‐3a* when treated with SpaC. These results suggest that there is a link between Caspase‐3−GSDMEa and Caspy2−GSDMEb pathways that were activated by SpaC in gut microbiota‐conserved zebrafish. However, SpaC can only activate Caspase‐3−GSDMEa pathway when the *caspy2* is knocked down. Therefore, it is likely that SpaC first activates Caspase‐3−GSDMEa pyroptosis, which leads to gut microbiota dysbiosis and outgrowth of LPS‐producing bacteria in the gut. And then LPS‐producing bacteria activate the Caspy2−GSDMEb pyroptosis pathway by releasing LPS to the intestinal environment. These results further demonstrated that the activation of Caspy2−GSDMEb pathway is a secondary consequence of Caspase‐3−GSDMEa pathway induced by SpaC. LPS can still activate the Caspy2−GSDMEb pathway even when *caspase‐3a* is knocked out. This result supports the importance of LPS‐producing bacteria and LPS production in the link between the two pyroptosis pathways induced by SpaC. In ZF4 cells, the importance of GSDMEa and GSDMEb in the SpaC and LPS‐inducing pyroptosis was revealed by the knockdown of the two pyroptosis executors, respectively. These results also suggest that no direct link exists between Caspase‐3−GSDMEa and Caspy2−GSDMEb pathways at the molecular level. Taken together, the gut microbiota plays an important role in intestinal pyroptosis induced by SpaC pilin of LGG. GSDMs are executors of Caspase‐dependent pyroptosis. Mammals, such mice, have a panel of pyroptotic gasdermins (GSDMs) (A−E) executing pyroptosis [49]. GSDMs can be activated by danger‐associated molecular patterns, pathogen‐associated molecular patterns (e.g., LPS) and double‐stranded DNA via inflammasome Caspase‐1/4/5/11−GSDMD pathways [50]. Although zebrafish only have a pair of GSDME (GSDMEa/b) [32, 33, 34, 35], this study suggests that the activation of LPS to GSDMEb is conserved among mammals and teleost. Besides, this study revealed the activation of Caspase‐3−GSDMEa pyroptosis pathway in zebrafish in vivo.

Generally, positive modulation of gut microbiota is an indicator of probiotic efficacy and is mostly characterized by the inhibition of the growth of harmful bacteria and stimulation of beneficial bacteria [51]. However, results from the current study inversely presented a case showing the negative modulation of gut microbiota of probiotics, through the action of the SpaC pilin of LGG. Furthermore, the expansion of Proteobacteria induced by SpaC demonstrated gut microbiota dysbiosis. Concerning this, Shin et al. (2015) revealed that increasing the abundance of Proteobacteria in the intestine is a signature of gut microbiota dysbiosis [52]. Petersen and Round (2014) characterized dysbiosis as loss of useful microbes, proliferation of pathogenic microbes, or loss of the diversity of the microbes [53]. Gut microbiota dysbiosis is commonly associated with hosts' diseases or disorders in humans [54, 55, 56, 57] and aquatic animals [58]. In the gut of *Litopenaeus vannamei* infected with the white spot syndrome virus, a significantly higher abundance of Proteobacteria compared with the healthy shrimp was observed [59]. Gut microbiota dysbiosis also occurs due to the exposure of the host to toxic environmental chemicals [60]. In this study, gut microbiota dysbiosis is the consequence of SpaC‐triggered IECs' pyroptosis of Caspase‐3−GSDMEa pathway. Therefore, gut microbiota dysbiosis can not only serve as an indicator of disease or health‐damaging status but also reflect the safety of exogenous stimuli. Based on our results, gut microbiota dysbiosis can act as an integrated indicator reflecting the safety of probiotics.

The specific pattern recognition receptors (PRRs) in the host intestine are responsible for the recognition of MAMPs [61]. SpaC is the adhesion factor of LGG as well as one kind of MAMP [38]. In mammals, the immunomodulation effect of SpaCBA depends on the activation of TLR2‐mediated signaling [11, 62, 63], and TLR2 recognizes LGG and initiates signaling pathways to coordinate immunity response [64]. Meanwhile, LGG cannot protect TLR2‐deficient mice from colitis induced by *Citrobacter rodentium* [65]. In mammals, TLR2 is involved in the recognition of MAMPs such as lipoprotein, lipoteichoic acid, peptidoglycan, and yeast zymosan. Generally, mammalian TLR2 forms heterodimers with TLR6 to sense lipoprotein and lipoteichoic acid [66]. However, the gene encoding TLR6 is absent in zebrafish genome [67]. Thus, zebrafish TLR2 may fail to sense and recognize LGG.

In this study, we used pull‐down assay conjugating LC−MS/MS technique to identify receptors that may interact with SpaC. Pull‐down assay is an in vitro technique to detect physical interactions between two or more proteins. This technique is an invaluable tool for confirming a predicted protein‐protein interaction [68]. Seven membrane receptors were screened by using pull‐down assay conjugating LC−MS/MS technique, including TLR4ba. Among these receptors, existing reports suggest that only TLR4 is reported to control pyroptosis in mammals [69, 70, 71, 72]. In line with these studies, the importance of zTLR4ba to SpaC‐induced pyroptosis was also validated by the knockdown of *TLR4ba* in zebrafish larvae. In line with our findings, the inhibition or activation of the TLR4 signal pathway can inhibit or activate intestinal pyroptosis in mammals [69, 70, 71, 72]. Therefore, the causal functional relationship between TLR4 signaling and cell pyroptosis is conserved among zebrafish and mammals. The two TLR4 components (TLR4a and TLR4b) of zebrafish are paralogous rather than orthologous to the hTLR4. Unlike hTLR4, zebrafish TLR4a/b has less sensitivity to LPS, the canonical ligand in humans. This is likely due to the evolution of TLR4a and TLR4b in zebrafish to provide alternative ligand specificities to the TLR immune defense system through the changes that occur in the extracellular portions [39, 73]. Although the ligands of zebrafish TLR4a/b have not been clarified [39], the interaction between SpaC and zTLR4ba revealed in this study suggests that SpaC may serve as a ligand to zTLR4ba. The zTLR4ba may be the PRR that recognizes SpaC and initiates pyroptosis pathways in the intestine of zebrafish. The different effects of SpaC on cell pyroptotic rate and activation of pyroptosis pathways also support that SpaC is a potential ligand of zTLR4ba but not hTLR4. Furthermore, the distinct ligand preferences of zTLR4ba and hTLR4 were discriminated against by their interaction with SpaC and their roles in subsequent pyroptosis activation. Taken together these results indicate that SpaC may be the species‐specific ligand to zTLR4ba of zebrafish. In our study, this was further validated by the strong binding affinity of SpaC and zTLR4ba. The shift of ligand recognition is likely due to the extracellular portions of zebrafish TLR4a and TLR4b, rather than the changes in the Toll/IL‐1 receptor (TIR) domains [39]. Accordingly, our results support the contribution of the extracellular domain of zTLR4ba in the species‐specific recognition of SpaC. Therefore, the species‐specific recognition of SpaC by zTLR4ba determines the host‐specificity of SpaC‐inducing intestinal damage.

It has been reported that surface pilus not only exist on LGG, but is also found on other probiotics, like *Bifidobacteria* and *Lactococcus lactis* [74, 75]. Though no damage to the intestinal surface or safety concerns was reported when using *Bifidobacteria* or *Lactococcus lactis* strains in livestock species [76, 77], feeding mandarin fish with the diet containing 10^10^ CFUs/kg *Lactococcus lactis* 3‐C‐18 resulted in negative effects on fish health [78]. These shreds of evidence suggest that application of nonhost probiotic may affect the health of the target animal through different mechanisms. For instance, the properties of pilus adhesion may influence the interactions between pilus adhesion and host cell membrane receptors [79], further contributing to the host specificity of probiotics.

## CONCLUSION

Taken together, this study reports for the first time the safety issues of probiotics from the perspective of species specificity. The SpaC pilin of LGG induces intestinal epithelium injury through a species‐specific activation of TLR4ba, which initiates Caspase‐3−GSDMEa pyroptosis and secondarily activates Gaspy2−GSDMEb pyroptosis via gut microbiota dysbiosis. This study may improve our understanding of the host‐specific beneficial/harmful mechanisms of probiotics. Future studies to test different *Lactobacilli* strains originated from other animal species are needed to draw more conclusive conclusions. To mitigate the potential risks of nonhost origin probiotics, we recommend two ways. The first one is to develop probiotic strains for the specific species rather than directly extend probiotic strains that are isolated from one species to another species. Secondly, gene editing technology will be useful to delete risk factors from a specific probiotic strain. Even though, this study presents a case of species‐specific probiotic safety issues, it is crucial to evaluate the effects of probiotics derived from nonhuman sources because these may threaten human health and should gain more attention. Although probiotics are generally regarded as nonpathogenic and safe to host, the safety issues of probiotics reported in humans still occur under certain specific circumstances [80, 81]. Probiotic application leads to systemic infections, deleterious metabolic activities and gastrointestinal side effects in these susceptible individuals [82]. These risks elicit the necessity of further research to describe the incidence and severity of adverse events related to probiotics [82]. The reported cases as well as our results suggest that the selection and application of probiotics should be cautious.

## METHODS

### Bacteria strain

*Lactobacillus rhamnosus* GG (ATCC53103) was purchased from the China Center of Industrial Culture Collection. The mutant strain PB22 of LGG was obtained from the Laboratory of Microbiology, Wageningen University and Research, Wageningen, Netherlands. Both LGG and PB22 strains were stationarity cultured in de Man, Rogosa and Sharp (MRS) broth at 37°C for 12 h. Then, the bacterial cells were harvested by using a refrigerated centrifuge (8000 rpm for 10 min at 4°C). The collected bacterial cells were washed three times with distilled PBS to remove MRS broth.

### Experimental animals

Gut microbiota‐conserved zebrafish (1‐month old, AB strain) were bred in the fish lab of Institute of Feed Research of Chinese Academy of Agricultural Sciences, Beijing, China. Gut microbiota‐conserved zebrafish were randomly assigned to acrylic tanks. The size of each tank was 20.0 cm × 14.0 cm × 10.0 cm. The density was 18 fish per tank. Keeping the rearing temperature at 25−28°C, the dissolved oxygen >6.0 mg/L, the pH value 7.0−7.5, the nitrogen content <0.50 mg/L, and the nitrogen content (as NO_2_) < 0.02 mg/L. Zebrafish were maintained at a 14:10 light/dark (L/D) cycle.

For *Lactobacillus* immersion, 1 month‐old zebrafish were maintained in water added with *Lactobacillus* strain (LGG or PB22, 10^10^ CFUs/mL) at a final concentration of 10^7^ CFUs/mL for Day 7 or 14, which was defined as the LGG or PB22 group. This concentration was referenced to He et al. [22] who reported that a 14‐day immersion of LGG at 10^7^ CFUs/mL can result to intestinal damage. phosphate‐buffered saline (PBS) buffer of equal volume was added into the tank water to serve as the Ctrl group. Four duplicate tanks per group were set. A total of 72 fish were used in each group (18 fish/tank). Zebrafish were fed with the basal diet (Table S2) twice a day (9:00, 17:00) to apparent satiation each time. For immersion of pilin subunits, 1‐month‐old zebrafish were maintained in water supplemented with recombinant SpaA, SpaB, or SpaC at a concentration of 10 μg/mL for Day 3, 7 or 14. Before immersion, zebrafish were netted into tanks gently and acclimated for 12 h. About three‐fourths water in each tank was changed daily. Fresh cultured *Lactobacillus* stains (10^10^ CFUs/mL) or prepared pilin subunits (stock concentration, 10 mg/mL) were added by volume ratio 1:1000 to maintain their final concentrations in the tank water. Four duplicate tanks per group were set. A total of 72 fish were used in each group (18 fish/tank). Zebrafish were fed with the basal diet (Table S2) twice a day (9:00, 17:00) to apparent satiation each time.

For the feeding trial, 1‐month‐old zebrafish were fed with the basal diet and experimental diets supplemented with SpaC (0.5 mg/g diet and 1.0 mg/g diet) or LPS (0.2 mg/g diet) (Table S2). The additive amounts of SpaC were calculated based on the average daily feed intake of zebrafish, as referenced to Plovier et al., 2017 [45]. The additive amount of LPS was set based on a previous study [83]. These zebrafish were defined as the Ctrl, SpaC0.5, SpaC1.0 and LPS0.2 groups. Three duplicate tanks per group were set. A total of 54 fish were used in each group (18 fish/tank). The feeding period continued for 3 weeks. Zebrafish were fed twice a day (9:00 and 17:00) to apparent satiation each time.

All procedures during immersion and feeding trials were conducted gently so that unnecessary damage or stress was avoided. Water quality was monitored every day during immersion and feeding trials as described above.

GF zebrafish were generated in the fish lab of the Institute of Feed Research of the Chinese Academy of Agricultural Sciences following established protocols [84, 85]. GF zebrafish were hatched in 30‐mL tissue culture bottles with a density of 20 zebrafish per bottle and were kept in an incubator with a constant temperature of 28°C and 14:10 L: D cycle. At 4‐day post‐fertilization (dpf), GF zebrafish were immersed with 10 μg/mL SpaC and 2 μg/mL LPS, respectively, or received gut microbiota derived from 1‐month‐old zebrafish fed with Ctrl, SpaC1.0 or LPS0.2. SpaC (stock concentration, 10 mg/mL) or LPS (stock concentration, 2 mg/mL) solution was added by volume ratio 1:1000 to maintain the final concentration in the water. The gut microbiota (10^8^ CFUs/mL) collected from 1‐month‐old zebrafish fed with one of the diets, was added to water to reach a final concentration of 10^6^ CFUs/mL. Three bottles containing a total of 60 GF zebrafish were used for each treatment (20 fish/bottle).

The *casp3a* ^−/−^ zebrafish (ZKO331) were purchased from the China Zebrafish Resource Center and maintained in the fish lab of Institute of Feed Research of Chinese Academy of Agricultural Sciences. Larvae of *casp3a* ^−/−^ zebrafish were hatched in hatching tanks. At 4‐dpf, *casp3a* ^−/−^ zebrafish larvae were assigned into 30‐mL tissue culture bottles using a pasteur pipette gently. The density was 20 zebrafish larvae per bottle. Then *casp3a* ^−/−^ zebrafish larvae were subjected to immersion of SpaC (10 μg/mL) and LPS (2 μg/mL), respectively. Similarly, SpaC (stock concentration, 10 mg/mL) or LPS (stock concentration, 2 mg/mL) solution was added by volume ratio 1:1000 to maintain the final concentration in the water. Three bottles of *casp3a* ^−/−^ zebrafish larvae were used for each treatment. A total of 60 *casp3a* ^−/−^ zebrafish larvae were used for each treatment (20 fish/bottle).

For experiments involving zebrafish larvae, all procedures during immersion were conducted gently so that unnecessary damage or stress was avoided. About three‐fourths water in each bottle was changed every 2 days using a pasteur pipette gently. Intestine of zebrafish larvae was isolated under stereo microscope.

### Cell lines

ZF4 cells (ATCC® CRL‐2050™) were established from a 1‐day old zebrafish embryo and cultured in Dulbecco's Modified Eagle Medium (DMEM)/Nutrient Mixture F‐12 medium containing 10% fetal bovine serum. ZF4 cells were cultured at 28°C in a humidified 5% carbon dioxide (CO_2_) and 95% air atmosphere. HEK293 cells (ATCC® CRL‐3216™) were cultured in DMEM containing 10% fetal bovine serum. Caco‐2 cells (ATCC® HTB‐37™) were established from the colon of human and cultured in Eagle's Minimum Essential Medium containing 10% fetal bovine serum. HEK293 and Caco‐2 cells were maintained at 37°C in a humidified 5% CO_2_ and 95% air atmosphere.

### Histopathological analysis

Hematoxylin and eosin (H&E) staining and transmission electron microscopy (TEM) analysis were used to measure the histomorphometry of the intestinal tract of zebrafish. For H&E staining, zebrafish intestines were rinsed with sterilized PBS, fixed in 4% paraformaldehyde in PBS, and then embedded in paraffin. Intestine sections prepared from the paraffin blocks were subjected to H&E staining. Images were obtained by Leica microscope. To assess the effects of different bacteria strains or pilins on intestinal morphology, images of intestine sections of each fish were randomly selected. The effects of different bacteria strains or pilins were monitored in terms of disorganized microvilli, edema or inflammatory infiltrate in lamina propria, vacuolar degeneration of IECs and cell shedding according to the previous studies [86, 87]. The morphological changes of each evaluation index were scored as follows: 0 = Normal, 1 = Slight disruption, 2 = Moderate disruption, and 3 = Severe disruption. Total histopathological scores of intestines were the sum of the scores for each evaluation index.

Tissue samples for TEM analysis were fixed with 2.5% glutaraldehyde. Intestinal specimens were examined using the TEM (Hitachi, H‐7500) at the Institute of Food Science and Technology, Chinese Academy of Agricultural Sciences, Beijing, China.

### Detection of serum LPS

The blood samples of the zebrafish were collected according to a previous study [88]. Serum LPS level was determined using a commercial ToxinSensor^TM^ Chromogenic limulus amebocyte lysate endotoxin assay kit (Genscript). The serum level of LPS in zebrafish was expressed as the endotoxin units per milliliter (EU/mL). All materials or diluents used for specimen collection and test reagent preparation were endotoxin‐free.

### Production of recombinant SpaC, SpaB, and SpaA proteins in *Escherichia coli*

The genomic DNA of LGG strain was extracted using a commercial DNA isolation kit (Tiangen) and then was used to amplify genes *spaC, spaB*, and *spaA*. Primers are listed in Table S5. The underlined sequences represent EcoR I and Xho I sites for cloning into the *p*EASY‐T3 vector to construct the plasmids of *spaC*‐T3, *spaB*‐T3, and *spaA*‐T3. Plasmids of *spaC*‐T3, *spaB*‐T3, *spaA*‐T3 and vector of *p*ET28a (+) were digested with EcoR I and Xho I. The gene *spaC*, *spaB* or *spaA* was inserted into the linearized *p*ET28a (+) vector with T4 ligation (New England Biolabs) to construct an expression vector. The *p*ET28a‐SpaC, *p*ET28a‐SpaB, or *p*ET28a‐SpaA vector was transformed into *E. coli* BL21 (DE3) chemically competent cells (Transgen). The bacteria were cultured in a lysogeny broth medium with kanamycin to a final concentration of 100 μg/mL at 37°C. When the optical density (OD) of the culture reached OD600  =  0.6−0.8, isopropyl‐β‐d‐thiogalactopyranoside (0.5 mM) was added to induce recombinant fusion protein production. After additional 12‐h incubation at 16°C, the bacterial cells were collected and lysed by a bugbuster protein extraction reagent (Merck‐millipore, Boston, US). The soluble protein was purified by the method of Ni‐NTA agarose chromatography (Wsac).

### Western blot analysis

Intestine tissues, zebrafish larvae or cells were lysed with ice‐cold radio immunoprecipitation assay lysis buffer supplemented with 1 mM phenylmethanesulfonylfluoride or phenylmethylsulfonyl fluorid and phosphatase inhibitors (Abcam, Cambridge, USA), and centrifuged at 12,000 rpm for 15 min at 4°C. Protein concentration was measured with bicinchoninic acid protein assay reagent (Pierce). First each protein sample were mixed with a 5× sodium dodecyl sulfate buffer according to a volume ratio of 4: 1, then bathing samples in boiling water for 10 min, and finally allow samples to cool to room temperature before applying to the gel. An equivalent amount of total protein was loaded into a 12% sodium dodecyl sulfate polyacrylamide gel for electrophoresis and then transferred to a polyvinylidene difluoride (PVDF) membrane with a pore size of 0.45 μm (Millipore). After blocking nonspecific binding with 5% skimmed milk in tris buffered saline with tween 20, the PVDF membrane was incubated with primary antibodies against glyceraldehyde‐3‐phosphate dehydrogenase (Sigma, SAB2708126, 1: 2000), zebrafish Caspase‐3 (Abcam, ab13847, 1: 1000), GSDMEa (1: 10000), Caspy2 (1: 10000), GSDMEb (1: 10000), Il‐1β (Abcam, ab9722, 1: 1000) or mammalian GSDME (Abcam, ab215191, 1:1000). The rabbit antibodies against zebrafish GSDMEa, Caspy2 and GSDMEb were all custom‐made in GenScript and gifted from the State Key Laboratory of Bioreactor Engineering, East China University of Science and Technology, Shanghai, China. The blots were developed using horseradish peroxidase‐conjugated secondary goat anti‐rabbit (CWBIO, CW0103S, 1:1,000) and the electrochemiluminescence‐plus system.

### Gut microbiota analysis

At the end of the 3‐week feeding trial, the gut contents of 1‐month‐old zebrafish were collected 4 h post the last feeding. The gut contents were collected under aseptic conditions. Each gut content sample was pooled from six fish. Bacteria DNA was extracted using the Fast DNA SPIN Kit for Soil (MP Biomedicals). The 16S V3−V4 region was amplified by using the primers as follows: 338F: 5′‐ACTCCTACGGGAGGCAGCA‐3′ and 806R: 5′‐GGACTACHVGGGTWTCTAAT‐3′. 16S rRNA gene sequencing was conducted at Biomarker Technologies using the illumina novaseq. 6000 platform (Illumina). Data analysis was performed using BMKCloud (www.biocloud.net). Primers that are set for universal bacteria or specific bacterial groups have been described in the scientific literature [89, 90, 91] and listed in Table S6. Results were expressed as Log_10_ copy numbers of bacterial 16S ribosomal deoxyribonucleic acid per milligram of intestinal contents.

### Prediction of gut microbiota function

The function of the gut microbiota based on the KEGG pathway of LPS biosynthesis at classification level 3 was predicted using the software of phylogenetic investigation of communities by reconstruction of unobserved states (PICRUSt2). Firstly, PICRUSt2 software was used to standardize the OTUs by comparing the species composition information obtained from 16S sequencing data. Then, combined with the KEGG family information corresponding to OTUs, the abundance of KEGG was calculated to analyze the functional differences between groups.

### Analysis of cell pyroptosis and apoptosis

Cell pyroptosis and apoptosis were detected using the commercial Annexin V‐fluorescein isothiocyanate (FITC) kit (Sigma, apoac). After SpaC or LPS treatment, ZF4 or Caco‐2 cells were collected and incubated with Annexin V−FITC and propidium iodide (PI) in the dark for 10 min at room temperature. The test was conducted using the Guava easyCyte Flow Cytometer (Merck Millipore). The fluorescence intensity was measured at an excitation wavelength of 488 nm using GREEN (525 nm) and RED (690 nm) filters. Data analysis was performed using FlowJo (version 10.8.1). Annexin V‐FITC stains apoptotic cells by recognizing the phosphatidylserine that is exposed on the external leaflet of the plasma membrane and also stains pyroptotic cells by recognizing phosphatidylserine on the inner leaflet due to membrane rupture [32]. The proportion of the dot plots in the upper right represented the cell pyroptotic rate (Annexin V positive, PI positive).

### Affinity pull‐down and LC‐MS/MS analysis

Pull‐down was performed following the instructions of a His‐protein interaction pull‐down kit (Pierce). First, recombinant His‐SpaC served as the bait protein and was immobilized to the Pierce Spin Column. Protein extracts from zebrafish intestines served as the prey proteins. Adding prepared prey proteins into the spin column containing the immobilized His‐SpaC, which was then incubated at 4°C for 12 h with gentle rocking motion on a rotating platform. Bait‐prey complexes were obtained after the elution by a gradient of 200 or 500 mM imidazole. The resulting bait‐prey complexes were digested using MS‐grade trypsin (Pierce) at 37°C overnight. Tryptic peptides were further purified by ZipTip (Millipore, ZTC18S096) and subjected to LC−MS/MS analysis on a Q Exactive mass spectrometer (ThermoFisher Scientific). Tandem MS data were extracted by the Proteome Discoverer software (Thermo Fisher Scientific, version 1.4.0.288) with the MASCOT searching engine version 2.3.02. The database used is the zebrafish UniProtKB/Swiss−Prot database (Release 2017‐11‐30, with 44,128 sequences).

### BiFC assay for direct visualization of the interaction between SpaC and zTLR4ba

BiFC assay was used to directly visualize protein‐protein interaction between SpaC and zTLR4ba in vitro. Cloning vectors for BiFC assay (*p*BiFC‐VN173, *p*BiFC‐VC155, *p*BiFC‐bJun‐VN173, and *p*BiFC‐bFos‐VC155) were obtained from GeneCopoeia, Inc. (Guangzhou, China). The coding regions of LGG SpaC (GenBank: FM179322.1, locus_tag = LGG_00444) and zebrafish TLR4ba (NCBI Reference Sequence: XM_009307228.3) were amplified by PCR and ligated into *p*BiFC‐VN173 and *p*BiFC‐VC155 (HA‐tag) vectors, respectively. HEK293 cells were cotransfected with *p*BiFC‐VN173‐SpaC and *p*BiFC‐VC155‐zTLR4ba fusion expression vectors using Lipofectamine™ 3000 Transfection Reagent (Thermo Fisher Scientific, Life Technologies). At 24 h posttransfection, the intensity and cellular location of the regenerated fluorescence signals were detected by a fluorescence microscope (Leica DMIL‐LED, Germany).

### Construction of plasmids *pc*DNA3.1‐zTLR4ba and *pc*DNA3.1‐zTLR4baEC‐hTLR4IC

TLR4 is composed of an extracellular domain containing leucine‐rich repeat (LRR) motifs, a transmembrane domain, and a cytoplasmic TIR homology domain [66]. To construct the plasmid of zTLR4ba, the gene sequence encoding zTLR4ba (NCBI Reference Sequence: XM_009307228.3) was cloned into the *p*cDNA3.1(‐) vector using the restriction sites NheI and HindIII. Meanwhile, the gene sequences encoding the extracellular domain of zTLR4ba, and the transmembrane and intracellular domains of hTLR4 (NCBI Reference Sequence: NM_138554.5) were cloned into the *p*cDNA3.1(‐) plasmid using the restriction sites NheI and HindIII, establishing a fusion‐expressing plasmid of zTLR4baEC‐hTLR4IC. The vector of *p*cDNA3.1(‐) was obtained from Detaibio, Inc. (Nanjing, China). Plasmid of *p*cDNA3.1‐zTLR4ba or *p*cDNA3.1‐zTLR4baEC‐hTLR4IC was transfected into Caco‐2 cells using Lipofectamine 3000 (Thermo Fisher Scientific, Life Technologies). Cells transfected with *p*cDNA3.1(‐) vector were used as the negative Ctrl. The transfected Caco‐2 cells were further treated with 10 μg/mL SpaC for an additional 24 h. Then cell death (apoptosis/pyroptosis) was determined by flow cytometry as described above.

### MST analysis

The encoding sequences of zTLR4ba extracellular domain (zTLR4ba, NCBI Reference Sequence: XM_009307228.3), hTLR4 extracellular domain (NCBI Reference Sequence: NM_138554.5) and hTLR2 extracellular domain (NCBI Reference Sequence: XM_017008573.1), were cloned into *p*ET30a vector using the restriction sites NheI and HindIII, constructing the expressing plasmids, respectively. The three plasmids were transformed into *E. coli* BL21 (DE3) chemically competent cells, respectively. The expression and purification of zTLR4ba, hTLR4, and hTLR2 were conducted as described above. Chemically synthesized oligonucleotides and *p*ET30a vector were obtained from Merrybio, Inc. (Nanjing, China). The interactions between SpaC and TLR proteins (zTLR4ba, hTLR4 and hTLR2) were measured using the Monolith NT.115 instrument (NanoTemper Technologies) at room temperature with 20% LED and 80% MST power. SpaC was labeled using Monolith Protein Labeling Kit Red‐NHS (CAT: MO‐L001). TLR proteins with serial dilution concentrations were prepared in PBS buffer and mixed with the labeled SpaC protein (30 nM), respectively. The mixtures were loaded onto premium capillaries (Monolith^TM^ NT.115 Series, NanoTemper Technologies). The fluorescence intensity signal due to thermophoresis was recorded using the red channel of the instrument. KD Fit of MO. Affinity Analysis v2.3 software (Nano Temper Technologies) was used to fit the curve and calculation of the dissociation constant (Kd). Assays were repeated with at least three biological replicates in triplicate.

### Quantitative real‐time PCR analysis

Total RNA was extracted from intestine and cell samples using Trizol Reagent (Cwbio). RNA was reversely transcribed to complementary DNA using FastKing gDNA Dispelling RT SuperMix (Tiangen). Quantitative real‐time PCR reaction (*q*RT‐PCR) was performed using SYBR® GreenSupermix (Tiangen). The results were stored, managed, and analyzed using LightCycler 480 software (Roche). The primer sequences are listed in Table S7.

### Gene knockdown

For zebrafish larvae, gene knockdown was conducted using morpholino oligonucleotides (MO) synthesized by Gene Tools (Philomath, OR). Zebrafish larvae were reared in embryo medium at 28°C to 4 dpf and then were allocated randomly to tanks using a pasteur pipette. The density was 80 larvae per 100 mL water per tank. The sequences of MO against zebrafish *caspy2* and *TLR4ba* were referenced to Yang et al. [33] and Zhang et al. [90], respectively. Zebrafish larvae were immersed with or without 10 μg/mL recombinant SpaC when treated with 100 nmol Ctrl MO, *caspy2* or TLR4ba morpholino for 3 d.

For ZF4 cells, gene knockdown was conducted by using *si*RNA designed and synthesized by GenePharma Co. Ltd. The sequence of negative Ctrl (scrambled *si*RNA, NC) and *si*RNA targeting zebrafish *gsdmea* and *gsdmeb* are listed in Table S7. ZF4 cells were first seeded on six‐well plates (Corning) and incubated for 24 h to subconfluence. Then the medium was removed and the cells were transfected with the scrambled *si*RNA or *si*RNA targeting *gsdmea* and *gsdmeb* using Lipofectamine RNAiMAX Transfection Reagent (Invitrogen, 13778930). The efficiency of the *si*RNA was determined by *q*RT‐PCR or immunoblotting 24 h posttransfection.

### Statistical analysis

All statistical analyses were performed in GraphPad Prism Version 8 (GraphPad Software) and ImageGP [92]. Variance homogeneity of the data was examined with Levene's test. Data involving more than two groups were analyzed using one‐way ANOVA followed by Duncan's test. Comparisons between the two groups were analyzed using the student's *t*‐test. Differences were considered significant at *p* < 0.05.

## AUTHOR CONTRIBUTIONS

Zhi‐Gang Zhou supervised and designed the research and gave conceptual advice for the manuscript. Zhen Zhang designed and conducted a major part of the experiments. Zhen Zhang and Qian‐Wen Ding wrote the manuscript. Qian‐Wen Ding completed partial cell experiments. Hong‐Ling Zhang assisted in intestine section assay, construction of various plasmids and pull‐down assay. Da‐Hai Yang provided primary antibodies that are essential for this study and advised on the manuscript. Qiang Hao, Hong‐Wei Yang, Shu‐Bin Liu and De‐Long Meng helped to perform western blot, gut microbiota analysis and cell culture. Le‐Luo Guan, Tsegay Teame, Chao Ran, Yuan‐Yuan Yao and Ya‐Lin Yang co‐analyzed and discussed the results. Willem Meindert Vos de provided the PB22 strain and gave advice for the manuscript. Chen‐Chen Gao performed the preparation of purified recombinant proteins. All authors have read the final manuscript and approved it for publication.

## CONFLICT OF INTEREST STATEMENT

The authors declare no conflict of interest.

## ETHICS STATEMENT

All procedures involving animal care and experiments were approved by the Institute of Feed Research of the Chinese Academy of Agricultural Sciences Animal Care Committee, under the auspices of the China Council for Animal Care (assurance No. 2017‐AF‐FRI‐CAAS‐001).

## Supporting information


**Figure S1:** SpaCBA pilus is responsible for the pro‐inflammatory response in zebrafish intestine
**Figure S2:** Indexes of gut microbiota composition and diversity.
**Figure S3:** Validation of efficiency of the gene knockdown.
**Figure S4:** Predictive interaction proteins obtained by affinity pulldown and LC‐MS/MS analysis.

**Table S1:** Scores of intestinal histology in zebrafish treated by LGG and PB22.
**Table S2:** Ingredients and proximate composition of diets for one‐month old zebrafish (g/100 g dry diet).
**Table S3:** Scores of intestinal histology in zebrafish treated by SpaC and LPS.
**Table S4:** Predictive interaction proteins obtained by affinity pulldown and LC‐MS analysis.
**Table S5:** The specific primers of the pilin subunits of LGG SpaCBA pilus.
**Table S6:** Primers for total bacteria or a specific phylotype.
**Table S7:** Sequences of primers and siRNA.

## Data Availability

Microbiota sequencing data in this study are available from the National Center for Biotechnology Information (NCBI) under accession number PRJNA1018345 (https://www.ncbi.nlm.nih.gov/bioproject/?term=PRJNA1018345). Supplementary materials (figures, tables, scripts, graphical abstract, slides, videos, Chinese translated version and update materials) may be found in the online DOI or iMeta Science http://www.imeta.science/.
