# Supplementary material for: Lactobacillus rhamnosus GG triggers intestinal epithelium injury in zebrafish revealing host dependent beneficial effects
Source: Imeta. 2024 Mar 25;3(2):e181. doi: 10.1002/imt2.181 (PMC11170971; doi:10.1002/imt2.181)

**Supporting information to: *Lactobacillus rhamnosus* GG triggers intestinal epithelium injury in zebrafish revealing host dependent beneficial effects**

**Running title**: LGG triggers intestinal injury in zebrafish

Zhen Zhang^1,2^, Hong-Ling Zhang^3^, Da-Hai Yang^4^, Qiang Hao^3^, Hong-Wei Yang^3^, De-Long Meng^3^, Willem Meindert de Vos^5,6^, Le-Luo Guan^2^, Shu-Bin Liu^3^, Tsegay Teame^3,7^, Chen-Chen Gao^3^, Chao Ran^1^, Ya-Lin Yang^1^, Yuan-Yuan Yao^1^, Qian-Wen Ding^1*^, Zhi-Gang Zhou ^3*^

^1^ Key Laboratory for Feed Biotechnology of the Ministry of Agriculture and Rural Affairs, Institute of Feed Research, Chinese Academy of Agricultural Sciences, Beijing, China

^2^ Faculty of Land and Food Systems, The University of British Columbia, Vancouver, Canada

^3^ China-Norway Joint Lab on Fish Gut Microbiota, Institute of Feed Research, Chinese Academy of Agricultural Sciences, Beijing, China

^4^ State Key Laboratory of Bioreactor Engineering, East China University of Science and Technology, Shanghai, China

^5^ Laboratory of Microbiology, Wageningen University and Research, Wageningen, Netherlands

^6^ Human Microbiome Research Program, Faculty of Medicine, University of Helsinki, Helsinki, Finland

^7^ Tigray Agricultural Research Institute, Mekelle, Tigray, Ethiopia

* Correspondence: zhouzhigang03@caas.cn (Zhi-Gang Zhou); dingqianwen@caas.cn (Qian-Wen Ding)

**Supplementary Materials**

**The file includes:**

Figure S1 to S4, and original full images of western blotting.


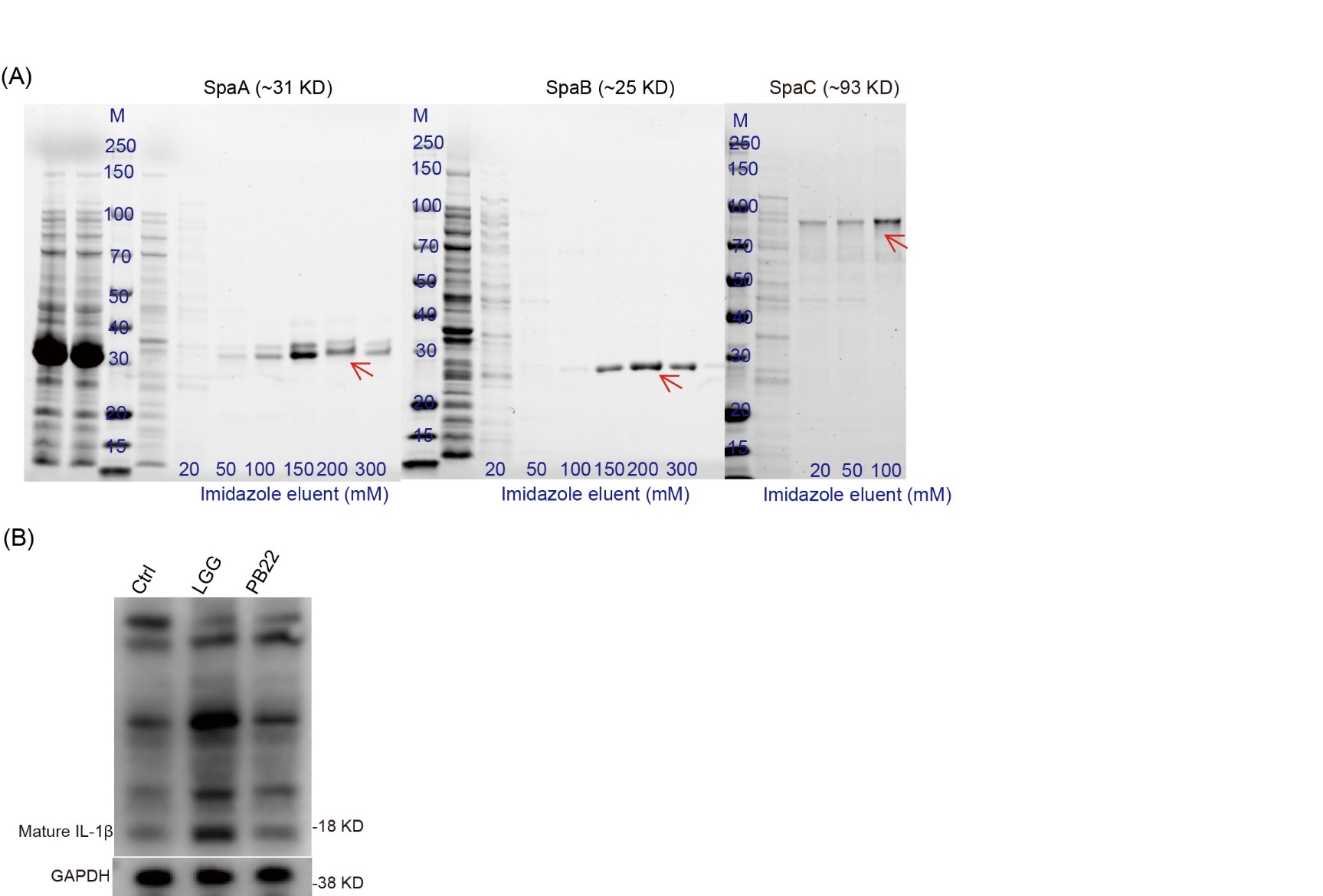


**Figure S1** **SpaCBA pilus is responsible for the pro-inflammatory response in zebrafish intestine.** (**A**) Recombinant SpaA, SpaB, and SpaC expression in *Escherichia coli*. (**B**) Release of mature IL-1β in the intestine of zebrafish immersed LGG or PB22 for 14 d. IL-1β, interleukin-1β.


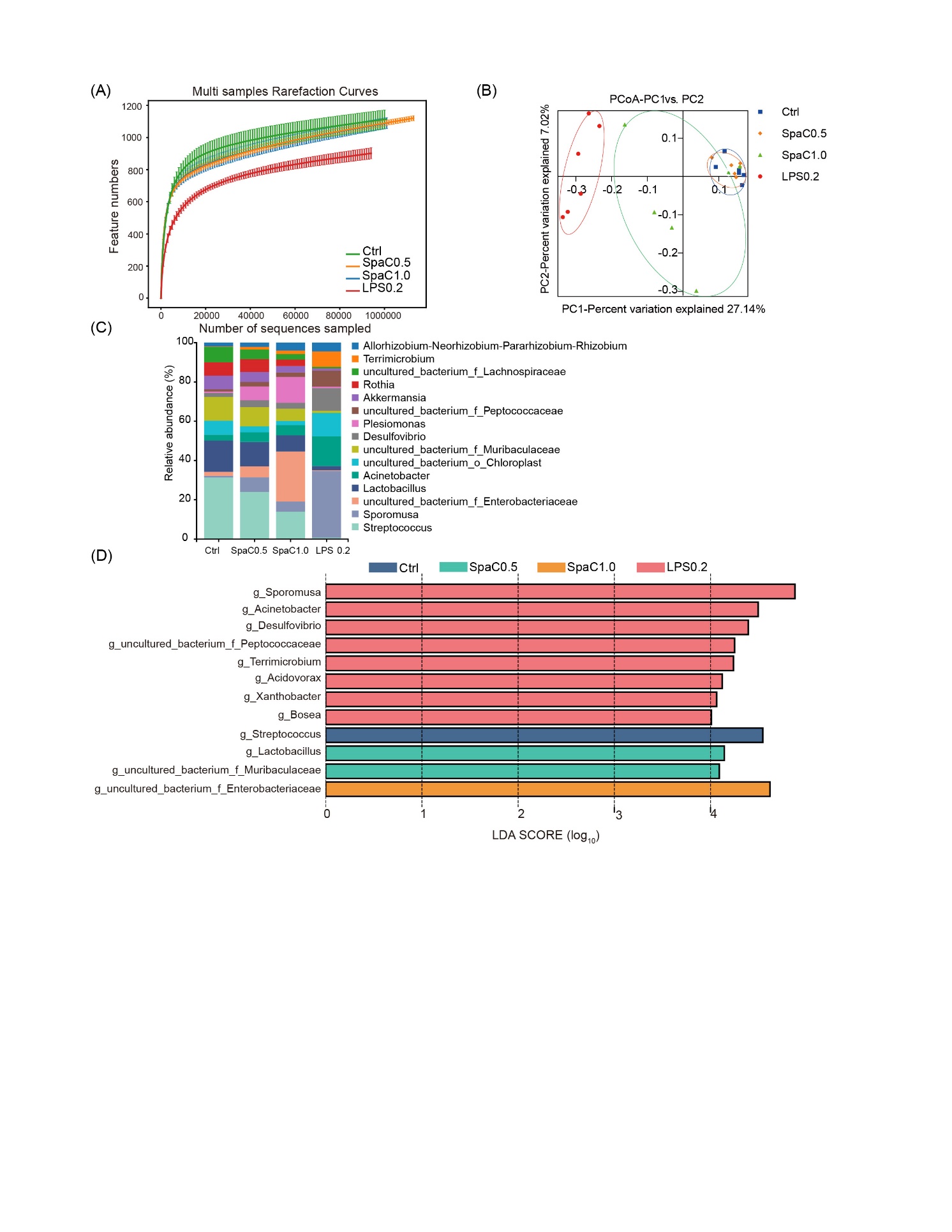


**Figure S2** **Indexes of gut microbiota composition and diversity**. (**A**−**D**) One-month-old zebrafish were fed with diets supplemented with 0.5 and 1.0 mg/g SpaC or diet supplemented with 0.2 mg/g LPS for three weeks (n = 6). (**A**) The rarefaction curves. (**B**) Principal coordinate analysis. (**C**) The composition and relative abundance of the top 15 abundant gut microbiota at genus level. (**D**) The linear discriminant analysis scores of 12 bacterial species of groups. LPS, lipopolysaccharide.


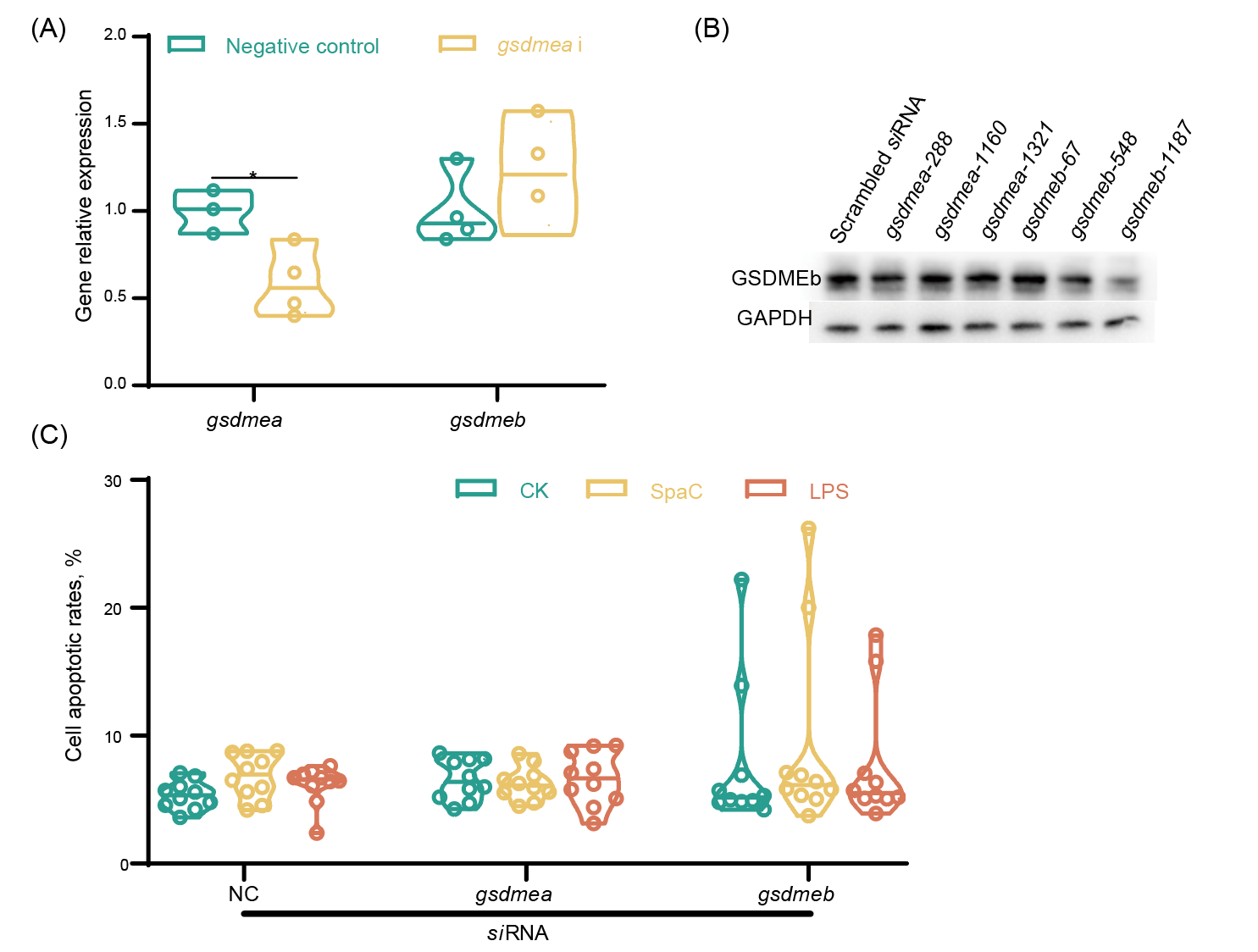


**Figure S3** **Validation of efficiency of the gene knockdown*.*** (**A**) Efficiency of the *si*RNA targeting *gsdmea* measured by *q*RT-PCR (*gsdmea*: *F* _1,5_ = 9.929; *gsdmeb*: *F* _1,6_ = 1.328; n = 3−4). (**B**) Efficiency of the *si*RNA targeting *gsdmeb* measured by immunoblotting. (**C**) Statistical apoptotic rates at 24 h post-treatment (Scrambled: *F* _2,27_ = 2.558; *gsdmea*: *F* _2,27_ = 0.134; *gsdmeb*: *F* _2,27_ = 0.194; n = 10). The Numbers of biologically independent samples are labeled on the violinplots. Horizontal line represents median in the violinplots. Statistics: Student’s *t*-test. *p* < 0.05, *. *si*RNA, specific small interfering RNA; GSDM, gasdermin; *q*RT-PCR, Quantitative real-time PCR reaction.


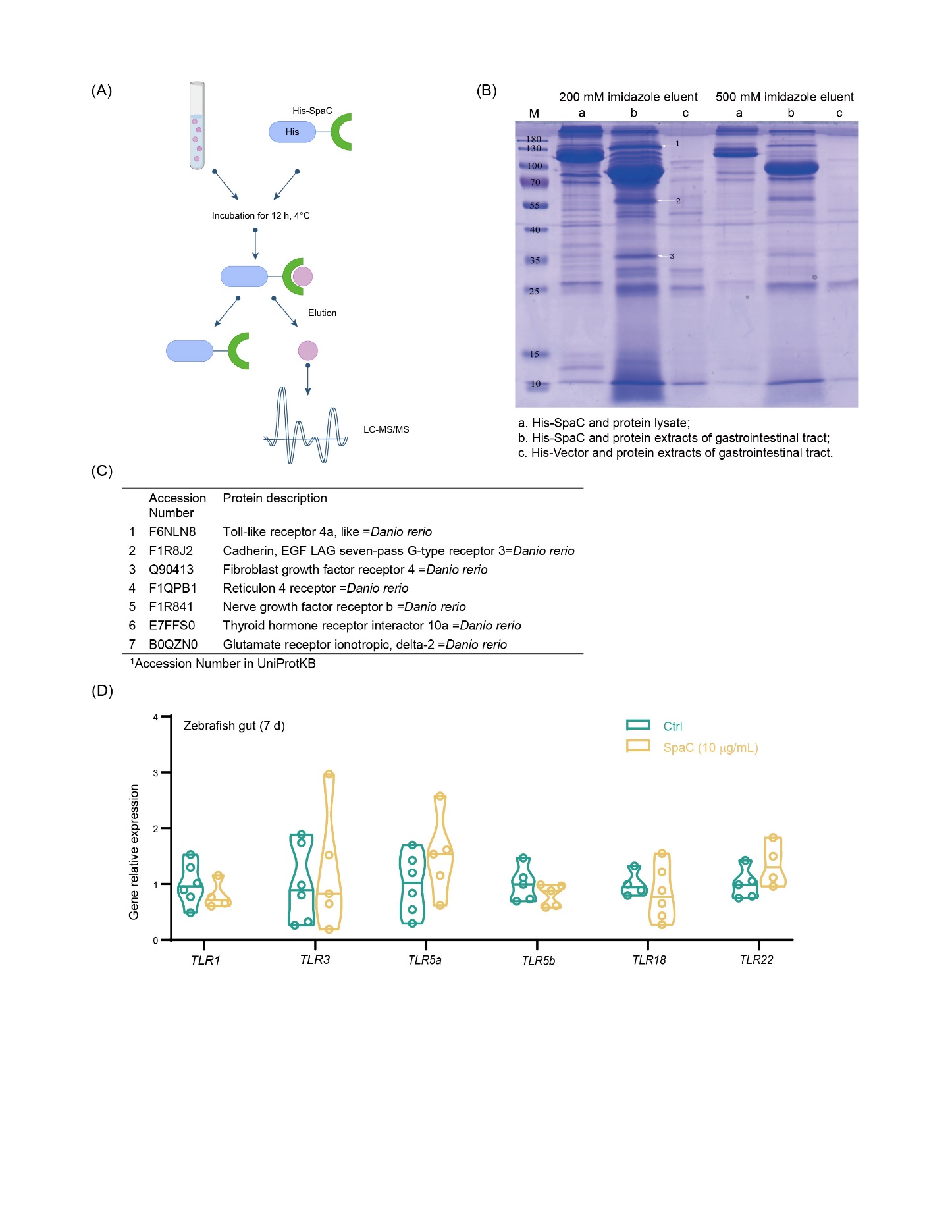


**Figure S4** **Predictive interaction proteins obtain by affinity pulldown and LC**−**MS/MS analysis**. (A) Illustration of detection of interaction proteins by pulldown and LC−MS/MS. (**B**) SDS−PAGE assay on bait−prey protein complexes (using a His-tagged SpaC protein as the bait protein). Bands 1, 2, and 3 were used for LC−MS/MS analysis. (**C**) Potential interaction membrane receptor proteins of SpaC obtained by pull-down assay. (**D**) One-month-old zebrafish were immersed with SpaC at 10 μg/mL for 7 d. Relative mRNA expression of genes encoding members of TLR family were measured by *q*RT-PCR (*TLR1*: *F* _1,8_ = 0.912; *TLR3*: *F* _1,9_ = 0.184; *TLR5a*: *F* _1,9_ = 1.734; *TLR5b*: *F* _1,8_ = 1.365; *TLR18*: *F* _1,8_ = 0.396; *TLR22*: *F* _1,7_ = 2.522; n = 4−6). The Numbers of biologically independent samples are labeled on the violin plots. Horizontal line represents median in the violin plots. Statistics: Student’s *t*-test. LC−MS, liquid chromatography−mass spectrometry; SDS−PAGE, sodium dodecyl sulfate-polyacrylamide gel electrophoresis; TLR, toll-like receptor.

**Original full image of western blotting**


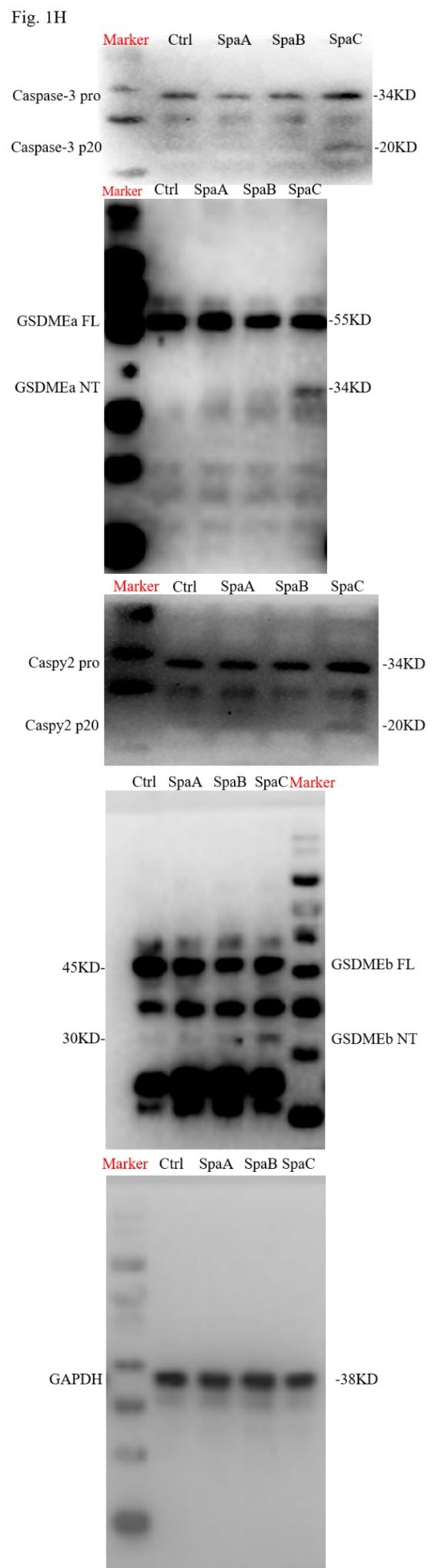

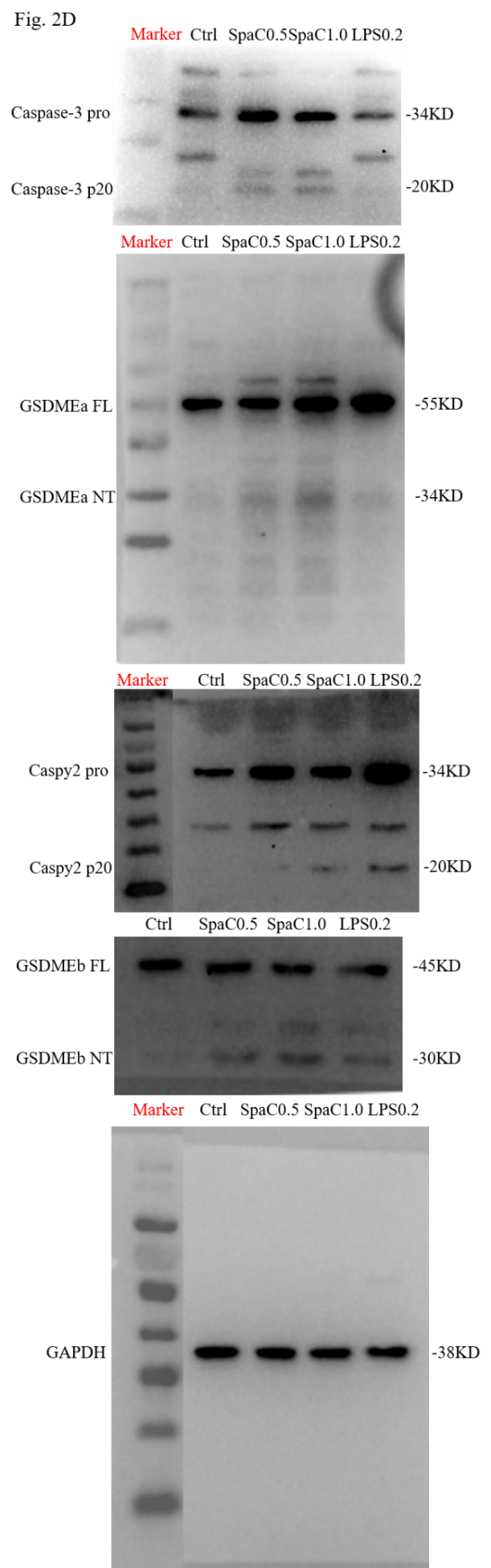

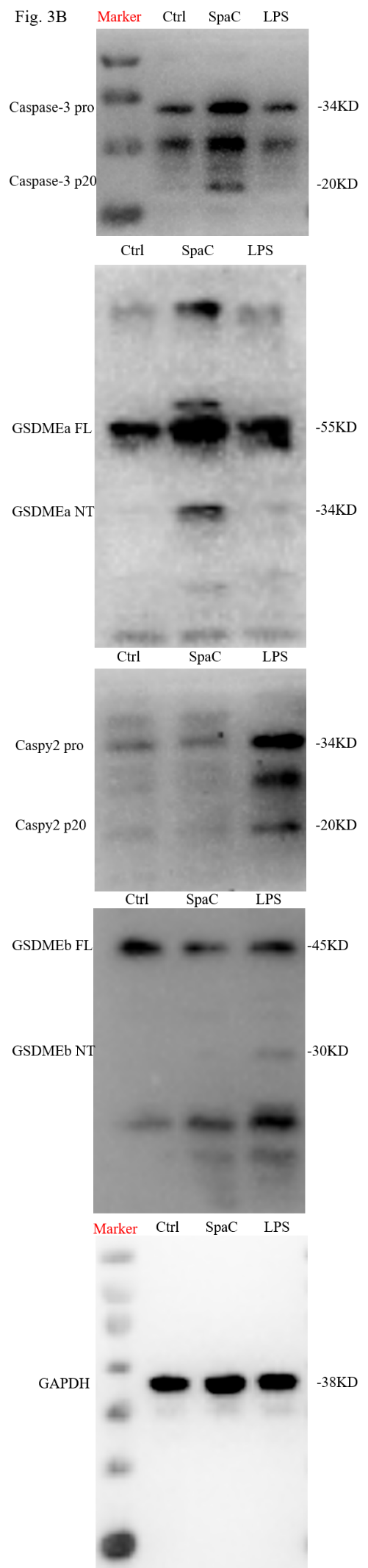

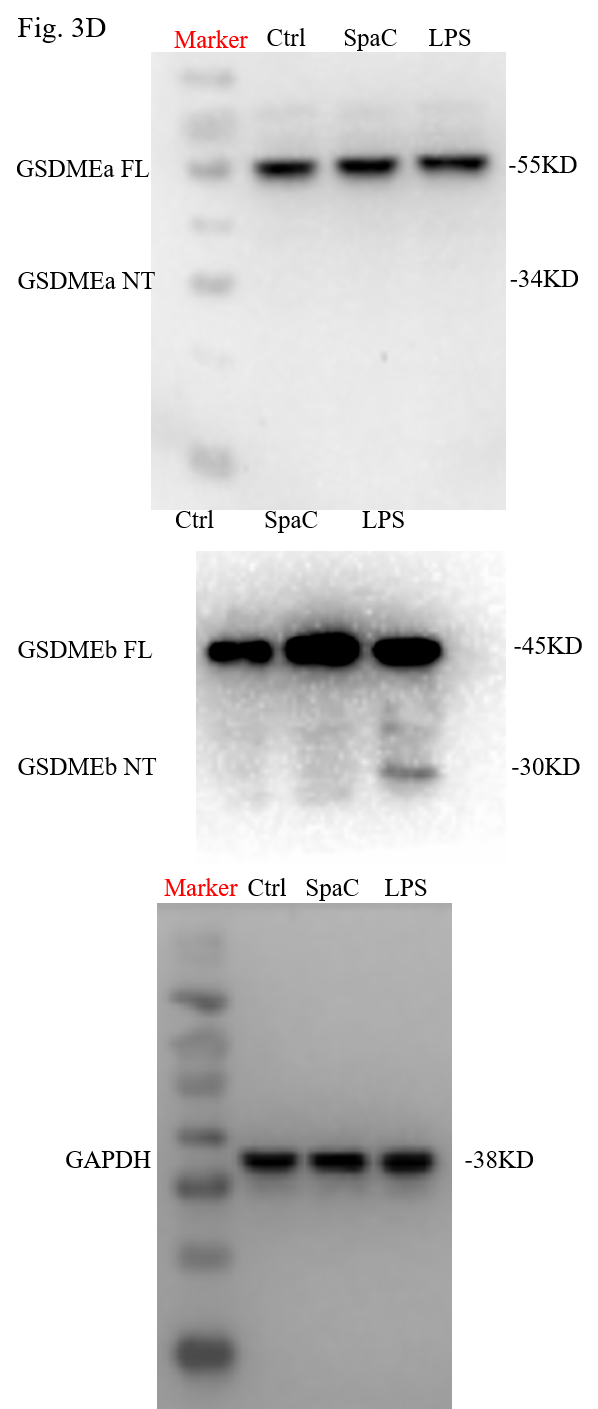

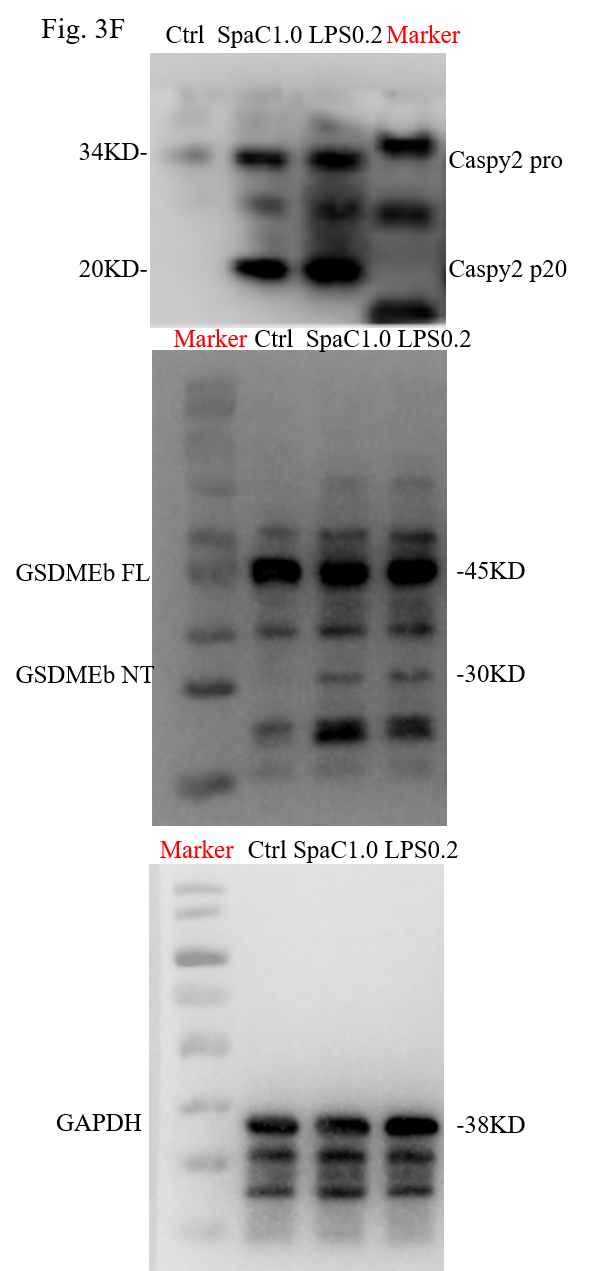

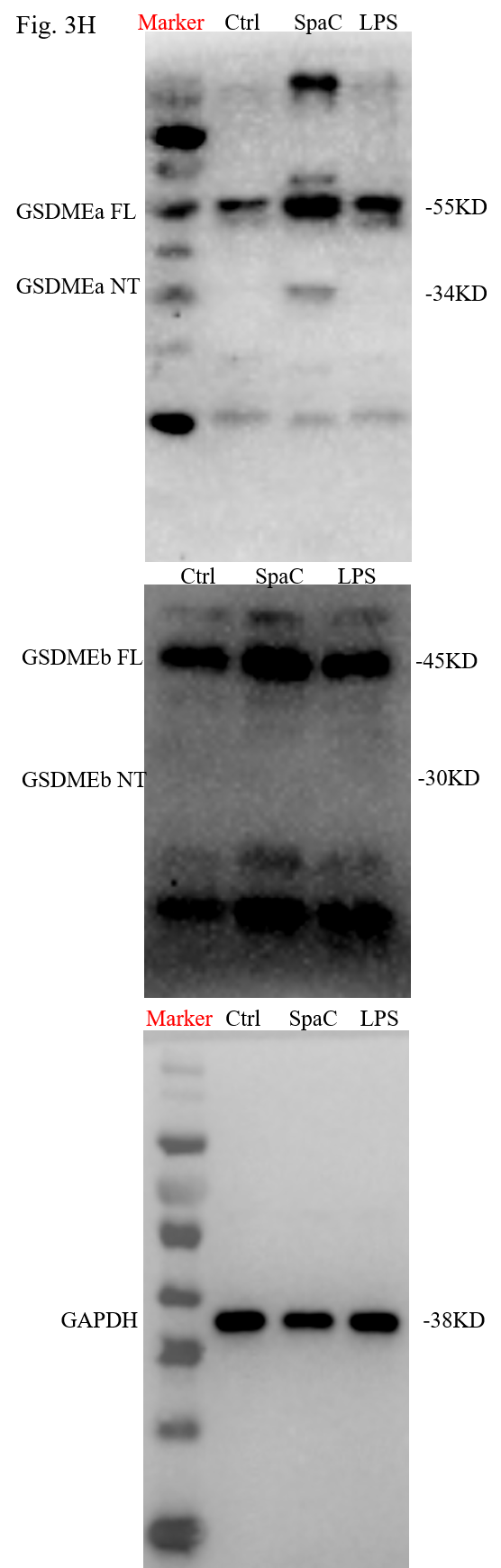

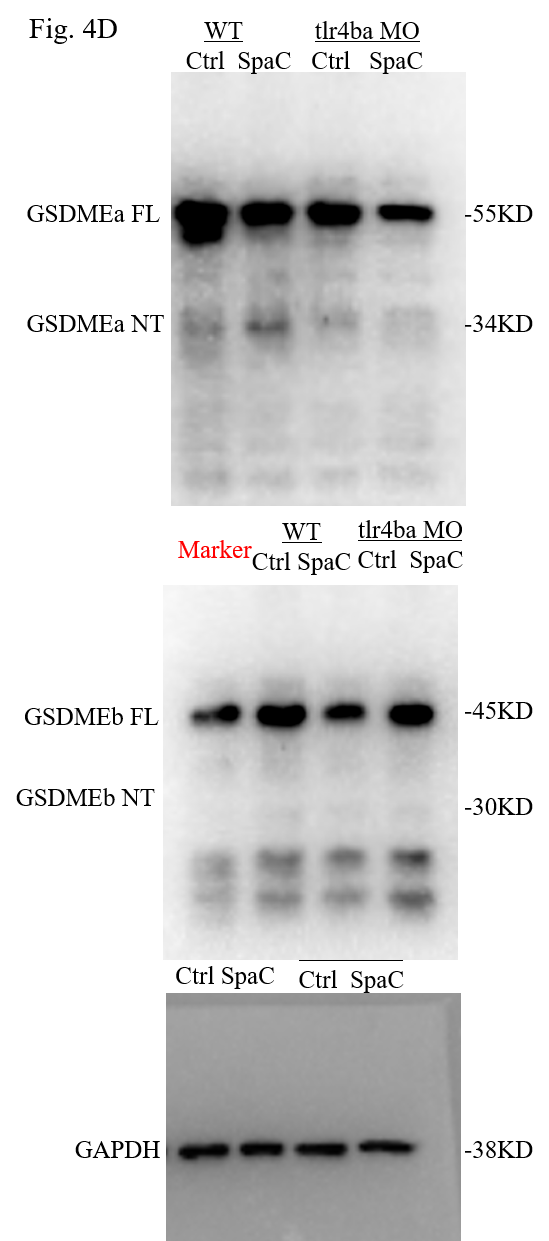

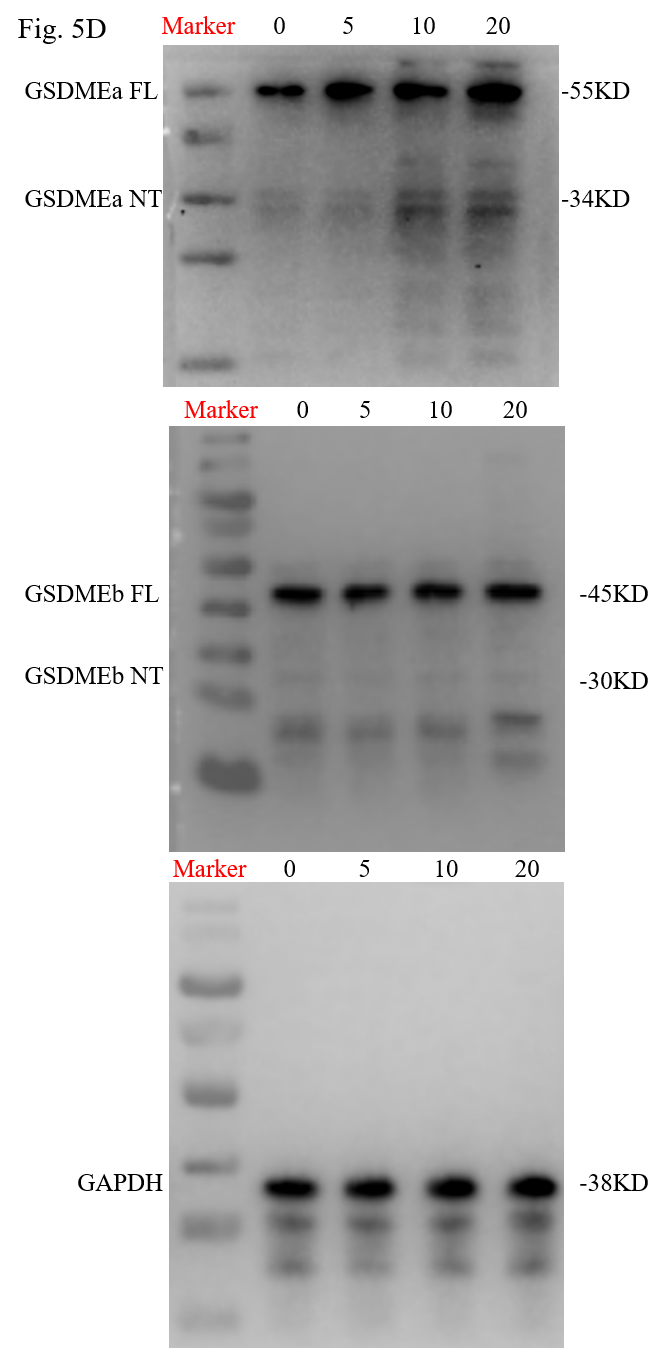

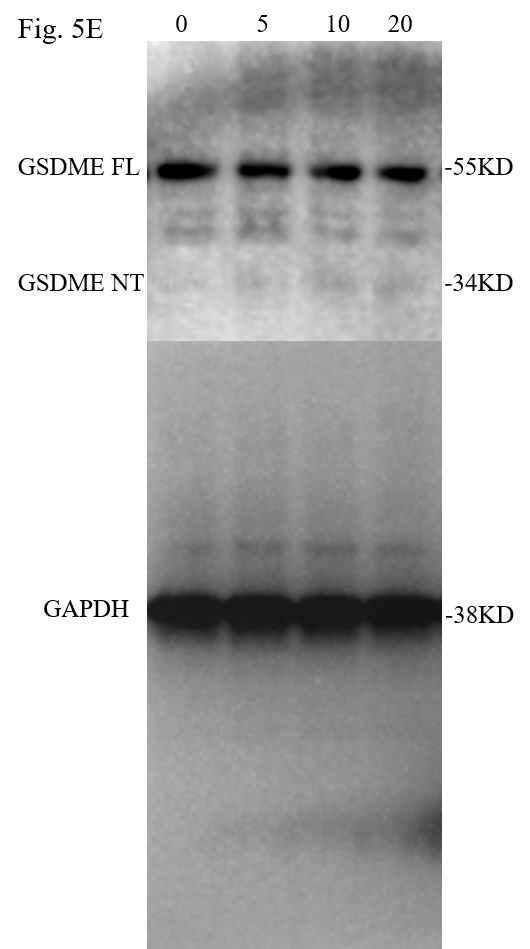

Supplement: Supplementary file 1 — Figure S1: SpaCBA pilus is responsible for the pro‐inflammatory response in zebrafish intestine Figure S2: Indexes of gut microbiota composition and diversity. Figure S3: Validation of efficiency of the gene knockdown. Figure S4: Predictive interaction proteins obtained by affinity pulldown and LC‐MS/MS analysis. [file IMT2-3-e181-s002.docx]
